# scATAnno: Automated Cell Type Annotation for Single-cell ATAC-seq Data

**DOI:** 10.1093/gpbjnl/qzaf108

**Published:** 2025-11-24

**Authors:** Yijia Jiang, Zhirui Hu, Feng Lu, Allen W Lynch, Junchen Jiang, Alexander Zhu, Ziqi Zeng, Yi Zhang, Gongwei Wu, Yingtian Xie, Rong Li, Ningxuan Zhou, Cliff A Meyer, Paloma Cejas, Myles Brown, Henry W Long, Xintao Qiu

**Affiliations:** Department of Medical Oncology, Dana-Farber Cancer Institute and Harvard Medical School, Boston, MA 02215, USA; The Center for Functional Cancer Epigenetics, Dana-Farber Cancer Institute, Boston, MA 02215, USA; Gladstone Institute of Data Science and Biotechnology, San Francisco, CA 94158, USA; Department of Medical Oncology, Dana-Farber Cancer Institute and Harvard Medical School, Boston, MA 02215, USA; The Center for Functional Cancer Epigenetics, Dana-Farber Cancer Institute, Boston, MA 02215, USA; Department of Data Science, Dana-Farber Cancer Institute, Boston, MA 02215, USA; Department of Biomedical Informatics, Harvard Medical School, Boston, MA 02115, USA; The Center for Functional Cancer Epigenetics, Dana-Farber Cancer Institute, Boston, MA 02215, USA; The Center for Functional Cancer Epigenetics, Dana-Farber Cancer Institute, Boston, MA 02215, USA; The Center for Functional Cancer Epigenetics, Dana-Farber Cancer Institute, Boston, MA 02215, USA; Department of Neurosurgery, Duke University, Durham, NC 27708, USA; Department of Biostatistics and Bioinformatics, Duke University, Durham, NC 27708, USA; Brain Tumor Omics Program, The Preston Robert Tisch Brain Tumor Center, Duke University, Durham, NC 27708, USA; Duke Cancer Institute, Durham, NC 27708, USA; Department of Medical Oncology, Dana-Farber Cancer Institute and Harvard Medical School, Boston, MA 02215, USA; Department of Medical Oncology, Dana-Farber Cancer Institute and Harvard Medical School, Boston, MA 02215, USA; The Center for Functional Cancer Epigenetics, Dana-Farber Cancer Institute, Boston, MA 02215, USA; Department of Medical Oncology, Dana-Farber Cancer Institute and Harvard Medical School, Boston, MA 02215, USA; The Center for Functional Cancer Epigenetics, Dana-Farber Cancer Institute, Boston, MA 02215, USA; Department of Medical Oncology, Dana-Farber Cancer Institute and Harvard Medical School, Boston, MA 02215, USA; The Center for Functional Cancer Epigenetics, Dana-Farber Cancer Institute, Boston, MA 02215, USA; Department of Data Science, Dana-Farber Cancer Institute, Boston, MA 02215, USA; Department of Biostatistics, Harvard T.H. Chan School of Public Health, Boston, MA 02115, USA; Department of Medical Oncology, Dana-Farber Cancer Institute and Harvard Medical School, Boston, MA 02215, USA; The Center for Functional Cancer Epigenetics, Dana-Farber Cancer Institute, Boston, MA 02215, USA; Department of Medical Oncology, Dana-Farber Cancer Institute and Harvard Medical School, Boston, MA 02215, USA; The Center for Functional Cancer Epigenetics, Dana-Farber Cancer Institute, Boston, MA 02215, USA; Department of Medical Oncology, Dana-Farber Cancer Institute and Harvard Medical School, Boston, MA 02215, USA; The Center for Functional Cancer Epigenetics, Dana-Farber Cancer Institute, Boston, MA 02215, USA; Department of Medical Oncology, Dana-Farber Cancer Institute and Harvard Medical School, Boston, MA 02215, USA; The Center for Functional Cancer Epigenetics, Dana-Farber Cancer Institute, Boston, MA 02215, USA

**Keywords:** scATAC-seq, Cell annotation, Uncertainty score, Single-cell epigenomics, Reference atlas

## Abstract

Recent advances in single-cell epigenomic techniques have increased the demand for single-cell assay for transposase-accessible chromatin using sequencing (scATAC-seq) analysis. One key analytical task is to determine cell type identity based on epigenetic data. Here, we introduce scATAnno, a Python package designed to automatically annotate scATAC-seq data using large-scale scATAC-seq reference atlases. This workflow generates reference atlases from publicly available datasets, enabling accurate cell type annotation by integrating query data with reference atlases without the use of single-cell RNA sequencing (scRNA-seq) data. To enhance annotation accuracy, we incorporated k-nearest neighbors (KNN)-based and weighted distance-based uncertainty scores to effectively detect cell populations within the query data that are distinct from all cell types in the reference data. We compared and benchmarked scATAnno against five other published cell annotation approaches, demonstrating its superior performance across multiple datasets and metrics. We further showcased the utility of scATAnno across multiple datasets, including peripheral blood mononuclear cells (PBMCs), triple-negative breast cancer (TNBC), and basal cell carcinoma (BCC), and demonstrated that scATAnno accurately annotates cell types across diverse biological conditions. Overall, scATAnno is a useful tool for scATAC-seq reference atlas construction and cell type annotation and can facilitate the interpretation of new scATAC-seq datasets in complex biological systems. scATAnno is publicly available at https://scatanno-main.readthedocs.io/.

## Graphical abstract

**Figure:**
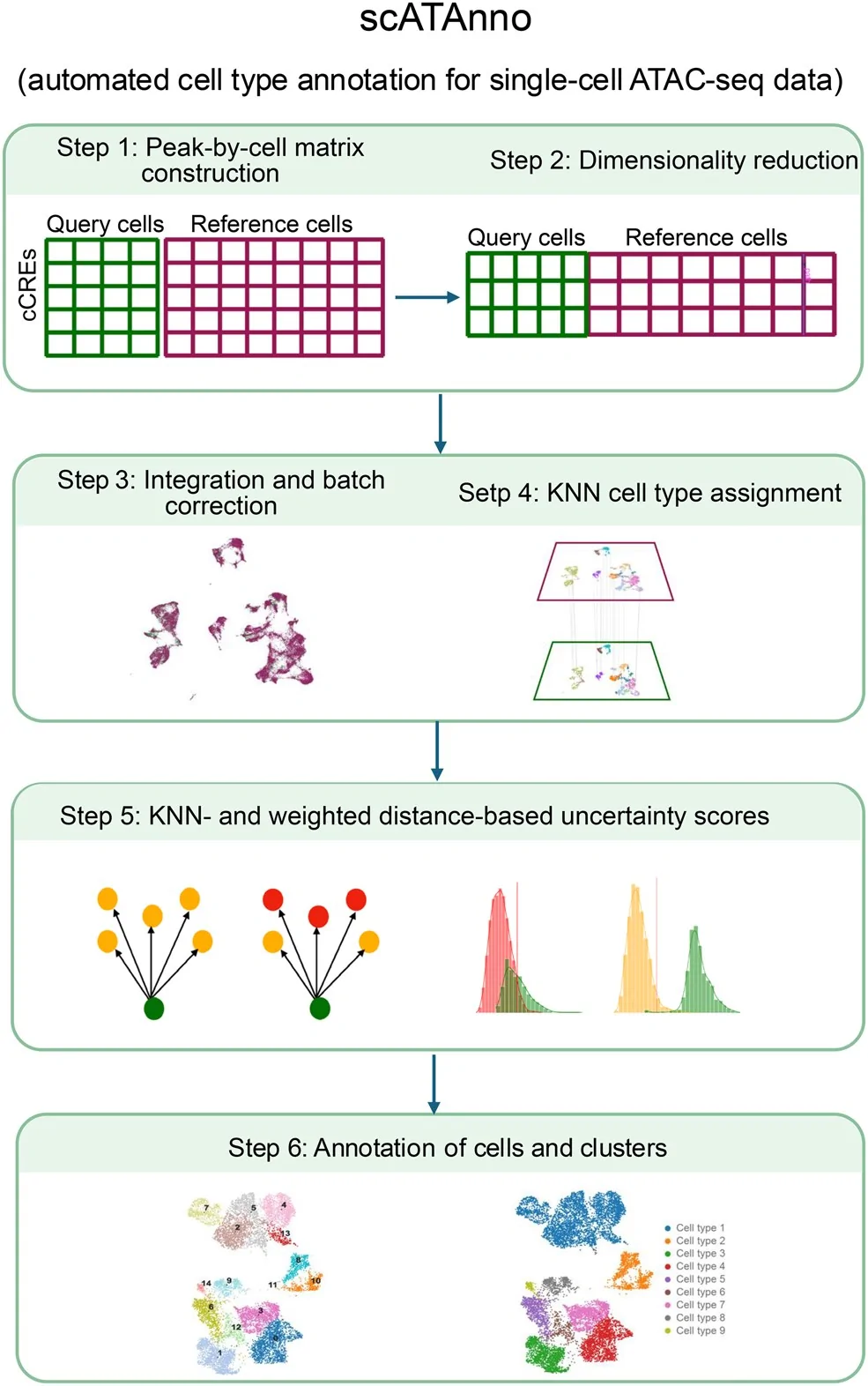

## Introduction

Recent advances in single-cell technologies have enabled the profiling of cellular heterogeneity in complex tissues at single-cell resolution [1–3]. Single-cell assay for transposase-accessible chromatin using sequencing (scATAC-seq) profiles chromatin accessibility in single cells and uncovers regulatory elements, known as *cis*-regulatory elements (CREs), which govern cell type-specific gene expression patterns [4–8]. Thus, scATAC-seq profiling plays an important role in understanding transcriptional patterns and chromatin configurations underlying cell states.

To identify what cell types are present in single-cell data, cell type annotation methods are needed. Many existing methods have been developed for single-cell RNA sequencing (scRNA-seq) data. These methods can be broadly categorized into two types: (1) leveraging well-annotated reference atlases to label cells in new datasets (known as query data) and (2) using a set of known gene markers to infer cell types. The first approach is used by tools such as SingleR, which predicts unannotated cells based on gene expression correlation with reference datasets [9,10], and scANVI, which is a deep generative model that transfers labels from reference to query data [11]. The second approach is exemplified by Garnett, which annotates cells based on predefined cell type-specific markers [12]. Although many methods have been designed for scRNA-seq data, only a limited number of tools are available for the automatic annotation of scATAC-seq data.

Single-cell chromatin accessibility data present unique challenges compared with scRNA-seq data. The first challenge is data sparsity and the high dimensionality of epigenomic features [13]. In contrast to scRNA-seq data, which typically contain one to several hundred transcripts per gene, scATAC-seq data often contain only a few fragment counts per peak because most cells contain only two copies of DNA at a given genomic locus, resulting in substantial data sparsity [4,5]. Moreover, scATAC-seq has much higher feature dimensions, as the number of potential genomic regulatory regions can reach millions, whereas the human genome contains only approximately 20,000 protein-coding genes [14]. Another challenge is the lack of marker enhancers for annotating scATAC-seq data. Although previous studies have profiled regulatory elements in tissues and cell lines [15], comprehensive catalogs of cell type-specific CREs remain incomplete [4,5]. Despite these challenges, cell type annotation at the scATAC-seq level remains important because the epigenetic landscape governs gene expression and plays a central role in defining cell identity. Most of the existing scATAC-seq annotation methods rely on converting peaks or genomic regions into gene activity scores and then employ various approaches to determine cell types. These approaches include (1) using scRNA-seq-based methods to predict cell types, such as Seurat (v3) [16], (2) utilizing supervised learning models trained on scATAC-seq reference to infer cell types in query data, such as Cellcano [17], and (3) assigning cell type labels based on gene markers, such as MAESTRO [17,18]. However, such methods rely on promoter and gene body regions as proxies for estimating gene activity scores, and therefore do not fully take advantage of hundreds of thousands of accessible enhancer regions distal to promoters in the scATAC-seq profile [19]. More recently, scATAcat [20] has been developed to annotate scATAC-seq cells at the pseudo-bulk level. There remains a need for methods that better utilize CREs in enhancer regions for cell type annotation at the single-cell level. The development of single-cell epigenomic techniques has also increased the availability of large-scale scATAC-seq datasets and a larger number of cell type-specific regulatory elements, spanning healthy adult cells to tumor-infiltrating lymphocytes (TILs) [21–23]. These scATAC-seq reference atlases collect data from multiple tissues and conditions, and enable the construction of peak references by aggregating robust signals from large numbers of single cells, providing an opportunity to automate cell type annotation for scATAC-seq data.

We present scATAnno, a Python-based workflow that directly and automatically annotates scATAC-seq data based on scATAC-seq reference atlases. Unlike many existing approaches, scATAnno directly uses peaks or CRE genomic regions as input features, eliminating the need to convert the epigenomic features into estimated gene activity scores. scATAnno tackles the high dimensionality of scATAC-seq data by leveraging spectral embedding to efficiently transform the data into a low-dimensional space. To create catalogs of CREs at single-cell level, scATAnno uses chromatin state profiles from a large-scale reference atlas to generate peak signals and reference peaks.

We show that the scATAnno workflow can generate scATAC-seq reference atlases from publicly available datasets generated using various scATAC-seq technologies, including 10x Genomics [21–23] and combinatorial indexing approaches [1,23,24]. Additionally, users can readily build customized scATAC-seq reference atlases using the scATAnno workflow (see Method). scATAnno enables accurate cell type annotation by integrating query data with reference atlases in an unsupervised manner. The results are further augmented with two uncertainty score metrics to quantitatively assess the confidence of cell type assignment. The first uncertainty score is based on the k-nearest neighbors (KNN) algorithm and determines if a single cell type is most similar to the query cell. The second uncertainty score is derived from a novel computation of the weighted distance between the query cell and reference cell type centroids in a low-dimensional space. This score determines if the most similar cell type is “close enough” to the query cell for confident assignment. We compared and benchmarked scATAnno against five other published cell annotation approaches, demonstrating its superior performance across multiple datasets and metrics. We further showcased the utility of scATAnno in multiple case studies, including peripheral blood mononuclear cells (PBMCs), triple-negative breast cancer (TNBC), and basal cell carcinoma (BCC), and demonstrated that scATAnno accurately annotates cell types and identifies previously unrepresented cell populations absent from the reference atlas. Furthermore, scATAnno enables efficient cell type annotation across tumor conditions and accurately annotates TILs in TNBC using a BCC TIL atlas. Overall, scATAnno is a cell type annotation tool specifically designed for scATAC-seq data and expedites our understanding of chromatin-based cellular heterogeneity in complex biological systems.

## Method

### Construction of reference atlases

The healthy adult reference atlas was obtained from the Gene Expression Omnibus database (GEO: GSE184462) [23]. To build a ready-to-use atlas for the scATAnno workflow, we obtained the count matrix provided by the original study, and selected deep-sequenced 1000 cells per minor cell type (total of 100,158 cells) and 890,130 adult-specific peaks. T cells, B cells, and myeloid cells were labeled as immune cells in the ready-to-use atlas.

The PBMC reference atlas was obtained from publicly available data [21]. To construct a ready-to-use atlas, we downloaded raw FASTQ files from BioProject (BioProject: PRJNA532774) to generate BAM files and fragment files. The original study encompassed both bone marrow cells and PBMCs. We excluded the bone marrow cells and selected 39,441 PBMCs. Fragment files were concatenated and filtered by these cell barcodes using the QuickATAC filter-barcodes function. Peaks were called on BAM files using MACS2 to generate a list of 196,618 reference peaks. Next, we used QuickATAC to intersect the fragment reads with reference peaks using the quick agg-countmatrix function (https://github.com/AllenWLynch/QuickATAC). We find that QuickATAC is unable to retain all reference peaks if there is an absence of reads within a peak location. To ensure that all reference peaks are generated in the final count matrix, we created a spike-in artificial fragment file that covered the maximum and minimum chromosomal coordinates of the reference peaks. We then concatenated the spike-in artificial fragment file and real fragment files to generate the input for the agg-countmatrix function. QuickATAC generated output files composed of MTX, barcodes, and feature CSV files. These files were imported into Python-based Scanpy to construct a peak-by-cell reference matrix in Anndata format, while retaining the entire peak space. We also examined chromatin accessibility across the *TRAV1-2*, *SLCA410*, and *PRSS35* genes, which are known as signature genes for mucosal-associated invariant T (MAIT) cells [25,26], in the reference atlas. The original gamma delta T cell annotation was corrected to MAIT cells. The BCC TIL atlas was constructed in a similar manner to the PBMC reference. We downloaded raw FASTQ files from the same BioProject (BioProject: PRJNA532774) to generate BAM files and fragment files of 17 BCC samples. We then selected 22,008 TIL cells whose cell barcodes were provided in the original study [21]. Fragment files were concatenated and filtered by these cell barcodes using QuickATAC filter-barcodes. A reference peak set (344,492 peaks) was generated by MACS2 calling on BAM files. To retain all reference peaks, we created a spike-in fragment file that covered the maximum and minimum genomic coordinates of the TIL peaks, and then concatenated the spike-in fragment and actual fragment files to construct a ready-to-use peak-by-cell BCC TIL reference matrix. Additionally, users can build their own reference atlases. A fragment file and a list of valid cell barcodes are required for reference building. To build a ready-to-use reference atlas, we first called peaks from the fragment file using the MACS2 peak-calling algorithm [21,27], and then constructed a peak-by-cell count matrix of the reference atlas using the QuickATAC agg-countmatrix function (https://github.com/AllenWLynch/QuickATAC). This reference-building process can be easily extended to other scATAC-seq datasets. scATAnno can also leverage Scanorama (https://github.com/brianhie/scanorama) to correct batch effects across datasets during reference construction. A full script of the reference-building steps can be found on GitHub (https://github.com/Yijia-Jiang/scATAnno-main/tree/main/prep_data/scATAnno-reference-building-example).

### Query data preparation

The data of PBMCs isolated through fluorescence-activated cell sorting (FACS) were downloaded from the GEO database (GEO: GSE123578). We downloaded the raw fragment files mapped to the hg19 reference genome from the GEO database, and lifted over the fragments mapping to the hg38 genome. The lymph node tumor of a patient diagnosed with B cell lymphoma was downloaded from 10x Genomics (https://www.10xgenomics.com/resources/datasets/). Ground truth labels were obtained from 10x Genomics (https://pages.10xgenomics.com/rs/446-PBO-704/images/10x_LIT000110_Data_Spotlight_Multiome_digital.pdf).

The original dataset contained low-quality cells and ambiguous cell type labels, such as mixed T and B cells. To prepare a clean dataset for benchmarking, we removed low-quality cells and curated cell type labels for B cells, tumor B cells, T cells, and monocytes.

The PBMC 10k data were directly downloaded from 10x Genomics (https://support.10xgenomics.com/single-cell-multiome-atac-gex/datasets/). To align the PBMC 10k features with the PBMC reference peaks, we concatenated the fragment file of PBMC 10k with the PBMC spike-in fragment file, and used the QuickATAC agg-countmatrix function to produce the MTX, barcodes, and feature CSV files. A final peak-by-cell matrix of query data was constructed using Scanpy [28]. The Seurat (v3) annotation was directly obtained by calling the “LoadData("pbmcMultiome", "pbmc.rna")” function in R, as shown in Vignette (https://satijalab.org/seurat/articles/atacseq_integration_vignette.html). We curated some cell type names from Seurat annotation to make the cell labels more comparable. Specifically, CD14^+^ and CD16^+^ monocytes were merged into monocytes, effector and central memory CD4^+^ T cells were merged into memory CD4^+^ T cells, and CD8^+^ effector memory T1 and T2 cells were merged into effector memory CD8^+^ T cells. The TNBC fragment file was obtained from the first author of the TNBC paper [22]. To align the TNBC features with the TIL reference peaks, we concatenated the fragment file of TNBC with the TIL spike-in fragment file and used QuickATAC agg-countmatrix and Scanpy to generate the peak-by-cell matrix of TNBC data. BCC fragment files were excluded from the BCC TIL reference building [21]. To align the BCC peaks with the TIL reference peaks, we concatenated the fragment file of BCC with the TIL spike-in fragment reads and used QuickATAC agg-countmatrix and Scanpy to generate the peak-by-cell matrix of BCC data. Optionally, QuickATAC provides quality control metrics, such as transcription start site (TSS) enrichment scores, which can be used to filter out low-quality cells.

### Dimensionality reduction

To reduce the high dimensionality of scATAC-seq data, we incorporated SnapATAC2 to perform spectral embedding [23,29]. The reference and query data were concatenated to form a combined peak-by-cell matrix. SnapATAC2 transforms the count matrix into a cell–cell similarity matrix using the Jaccard similarity score, which computes the ratio of the intersection and the union of two cells to estimate the similarity between cells [29]. The Jaccard similarity matrix is subsequently normalized to regress out the sequencing depth bias. Next, it computes the normalized Jaccard similarity matrix, followed by eigenvector decomposition to obtain the low-dimensional components. To reduce the computational cost of analyzing high-dimensional data, SnapATAC2 first samples “landmark” cells that capture the data distribution of the original points, and obtains the low-dimensional components of these highly informative cells. Finally, it projects the remaining cells into the same low-dimensional components and obtains low-dimensional components for all cells [5,29].

### Batch correction

To account for the potential technical variability of the reference atlas and query data, we applied Harmony for batch correction to eliminate potential batch effects [30]. Harmony was applied to low-dimensional embeddings of the concatenated reference and query datasets to regress out batch effects. The harmonized embeddings more accurately captured the true biological signals shared between the reference atlas and query data.

### Cell type assignment

In the initial cell type assignment, each query cell was assigned a cell label using its closest KNN in the reference atlas, based on the low-dimensional embeddings. The KNN algorithm is implemented using the Scikit-learn package in Python [30,31].

### Uncertainty score measurement

Suppose$\;X^{R}=(x_{ic}^{R},\;i=1,...,q;\;c=1,\ldots ,n)$ is a q×n matrix representing the coordinates of n reference cells in the latent space, where each column corresponds to a cell and each row corresponds to a coordinate.$\;X^{Q}=(x_{ij}^{Q},\;i=1,...,q;\;j=1,\ldots ,m)$ is a q×m matrix representing the coordinates of m query cells.$\;y^{R}=\;{\left(y_{1}^{R},...,y_{n}^{R}\right)}^{T}$ is a n-dimensional vector, where each $\;y_{c}^{R}\in \{1,\ldots ,K\}\;$is the cell type label of each reference cell.$\;y^{Q}=\;{\left(y_{1}^{Q},...,y_{m}^{Q}\right)}^{T}$, a m-dimensional vector, where each$\;y_{j}^{Q}\in \{1,\ldots ,K\}\;$is the predicted cell type label of query cell *j*.

### KNN-based uncertainty score

In the first step, we compute the uncertainty score of cell type assignment of each query cell. For a query cell j, we identify the p nearest reference cells ($N_{𝒿}$) based on the Euclidean distance in the low-dimensional embeddings, *i.e.*,$\;\mathrm{dist}(x_{j}^{Q},\;x_{c}^{R})$ =$\;{\left|\left|x_{j}^{Q}-\;x_{c}^{R}\right|\right|}_{2},\;c\in N_{𝒿}$. We compute the standard deviation of these nearest distances:$\;sd_{j}=\sqrt{\sum_{c\in N_{𝒿}}{\mathrm{dist}(x_{j}^{Q},x_{c}^{R})}^{2}/p}$. Then, we apply a Gaussian kernel function to transform the distance into a similarity measurement:$\;S(j,c)=\exp(\frac{-\text{dist}(x_{j}^{Q},x_{c}^{R})}{{\left(2/sd_{j}\right)}^{2}}),\;c\in N_{𝒿}$. Finally, we compute the probability of assigning a cell type to each query cell:$\;P(y_{j}^{Q}=k|x_{j}^{Q})=\frac{\sum_{c\in N_{𝒿}}S(j,c)I(y_{c}^{R}=k)}{\sum_{c\in N_{𝒿}}S(j,c)}$. Then, the uncertainty score is$\;u_{j,k}=1-P(y_{j}^{Q}=k|x_{j}^{Q})$. Based on this score, we can assign cell types as follows:


$$\hat{y}=\arg\underset{k}{\min}u_{j,k}$$

(1) yjQ^= {y^  if uj,y^< 0.5 Unknown  Otherwise

and the uncertainty score for cell j in the first step is:$\;u_{j}^{1}=\underset{k}{\min}u_{j,k}$

In the KNN-based uncertainty score, a query cell will be assigned a low uncertainty score if its k-nearest neighbors belong to the same reference cell type or a high uncertainty score if its k-nearest neighbors belong to mixed reference cell types.

### Weighted distance-based uncertainty score

The goal of computing the weighted distance-based uncertainty score is to measure the distance between each query cell and the cell type assigned to it, with the intuition that a query cell far away from the assigned cell type cluster is likely to be an unknown cell. The computation of the weighted distance-based uncertainty score consists of two steps: (1) computing the weights of low-dimensional embeddings for a specific cell type *k* to capture the relative importance in discriminating cell type *k*, and (2) comparing within-cell-type reference distance and the weighted distance between each query cell and the centroid of its assigned cell type *k*.

In the first step, we compute the weight of each latent dimension to distinguish each cell type from the rest, based on the assumption that low-dimensional components have different discriminatory power for a specific cell type. For cell type *k*, the weight of each dimension i, denoted as$\;W_{ik}\;\;\mathrm{for}\;i=1,\ldots ,q$, is defined as the ratio of the average between-cell-type distance to the within-cell-type variation, which is computed using *n* reference cells as follows:


(2) $$\tilde{w_{ik}}=\frac{d_{i,-k}}{d_{i,k}}=\sum_{c=1}^{n}\sqrt{{\left(x_{ic}^{R}-\bar{x_{i,-k}}\right)}^{2}}I(y_{c}^{R}=k)/\sum_{c=1}^{n}\sqrt{{\left(x_{ic}^{R}-\bar{x_{i,k}}\right)}^{2}}I(y_{c}^{R}=k)$$

where$\;\bar{x_{i,k}}\;$is the robust centroid of cell type *k* after removing outlier cells, and$\;\bar{x_{i,-k}}\;$is the robust centroid of the rest of the cells except cell type *k*. Specifically, we first identify the centroid of cell type *k* and the centroid of the remaining cells in the *i*-th embedding. We first remove the outlier cells based on Uniform Manifold Approximation and Projection (UMAP) to construct a cleaner structure of reference atlas and then compute the weights. We compute the within-cell-type distance *d*(*i*,*k*), representing the distance between the reference cells of cell type *k* and the reference centroid of cell type *k*, and the between-cell-type distance *d*(*i*,-*k*), representing the distance between the reference cells of cell type *k* and the centroid of the remaining cell types (Equation 1). The ratio of the average between-cell-type distance to the within-cell-type distance defines the weight of embedding *i* for cell type *k*. We repeat the process to get the weights of all latent embeddings for cell type *k* and then normalize the weights.$\;W_{ik}\;$is higher if reference cells are closer to their respective cell type centroid and far away from the other cell types. And embeddings assigned with high weights are more important to distinguish a given cell type. Then, we normalize$\;\tilde{w_{ik}}\;$for each cell type as follows:


(3) $$w_{ik}=\frac{\tilde{w_{ik}}}{\sum_{i=1}^{q}\tilde{w_{ik}}}$$

In the second step, based on these weights, we compute the within-cell-type weighted distance for reference cells and compare the distance of each query cell to these reference distances. If the distance of the query cell is significantly larger than the distance of reference cells, we reject the annotation of the query cell. For query cell *j* with a predicted cell type label$\;\hat{y_{j}^{Q}}=k$, we compute the weighted distance for each reference cell in cell type *k* and for the query cell as follows:


(4) $$\text{dis}t_{W}(x_{c}^{R},\bar{x_{k}})\;=\;\sum_{i=1}^{q}w_{ik}\sqrt{{\left(x_{ic}^{R}-\bar{x_{i,k}}\right)}^{2}},\;\forall c\;y_{c}^{R}=k$$

(5) $$\text{dis}t_{W}(x_{j}^{Q},\bar{x_{k}})\;=\;\sum_{i=1}^{q}w_{ik}\sqrt{{\left(x_{ij}^{Q}-\bar{x_{i,k}}\right)}^{2}}$$

Then, we compare the weighted distance of the query cell to the 95% quantile of all reference cells with cell type *k*. If the query distance is greater than the 95th percentile of the within-cluster distance of cell type *k*, we set the uncertainty score to 1.0 and annotate the cell as unknown:


(6) yjQ^= {  k  if distW(xjQ,xk¯) < distW(xcR,xk¯)0.95Unknown  Otherwise

The uncertainty score for cell *j* in the second step is:


(7) uj2= {0  if distW(xjQ,xk¯) < distW(xcR,xk¯)0.951  Otherwise

The distance-based uncertainty score assesses whether a query cell is positioned far from the centroid of its reference cell type. This score is calculated based on the distance between the query cell and the reference cell centroid, as well as the overall distribution of the reference cells.

We summarized the advantages, limitations, and examples in **Table 1** to provide a clearer comparison of the two uncertainty scores in scATAnno.

**Table 1:** Comparison of two uncertainty scores

	KNN-based uncertainty score	Weighted distance-based uncertainty score
Advantage	Consider whether the k-nearest neighbors belong to the same reference cell type, capturing local neighborhood consistency	Evaluate the distance between the query cell and the centroid of the reference cell type, providing a global measure of uncertainty
Limitation	Cannot determine if query cells are genuinely close to reference cells, as it relies only on the nearest neighbors	Do not consider whether the surrounding cells belong to the same cell type, potentially missing the local context
Challenging case	If a query cell is far from all reference cells but its nearest neighbors belong to the same type, KNN-based uncertainty may fail to detect uncertainty; the weighted distance-based uncertainty score can be helpful in this case	If a cell is positioned between two closely related cell types, only the distance to the centroid is considered, ignoring local cell labels; the KNN-based uncertainty score can be helpful in this case

*Note*: KNN, k-nearest neighbors.

### Cluster-level annotation

If no prior cluster information is provided, we cluster query cells using the Leiden algorithm to assign similar cells into the same group based on low-dimensional embeddings. The Leiden algorithm is implemented using scanpy.tl.leiden and applied to the query data with high resolution [28,32]. We determine the cell type of each cluster by taking the most abundant cell type. If cluster information is provided by users, scATAnno uses the cluster information and assigns the cluster-level annotation by majority voting, *i.e.*, taking the most abundant cell type. In this way, all query cells are annotated at the cluster level.

### Rare cell type annotation

Recognizing rare cell types is indeed a challenging aspect of cell annotation, and scATAnno incorporates several strategies to address this effectively.

First, scATAnno leverages spectral embedding. Spectral embedding utilizes similarity scores between data points as its foundation. Even for rare cell types with only a few data points, their distinct similarity scores allow them to stand apart from other cell types in the spectral space, making them easier to identify.

Second, during the reference-building process, we balance the cell numbers across different cell types to ensure that rare cell types are well-represented. This approach helps maintain the integrity of the reference and enhances its ability to identify rare cell populations.

Lastly, scATAnno includes two uncertainty scores to handle cases in which rare cell types may be absent from the reference. These scores can flag such cells as “unknown”, providing a mechanism to capture rare populations that are not explicitly present in the reference.

These combined strategies contribute to scATAnno’s robust ability to recognize rare cell types.

### Coverage plots of scATAC-seq data

Coverage plots were generated using Signac [26]. First, the fragment index file was generated from the fragment file for each query dataset. The fragment file was then read into Signac, and the accessibility of peaks over genomic regions was plotted using the CoveragePlot function in Signac [26].

### The scATAnno workflow

**Figure 1:**
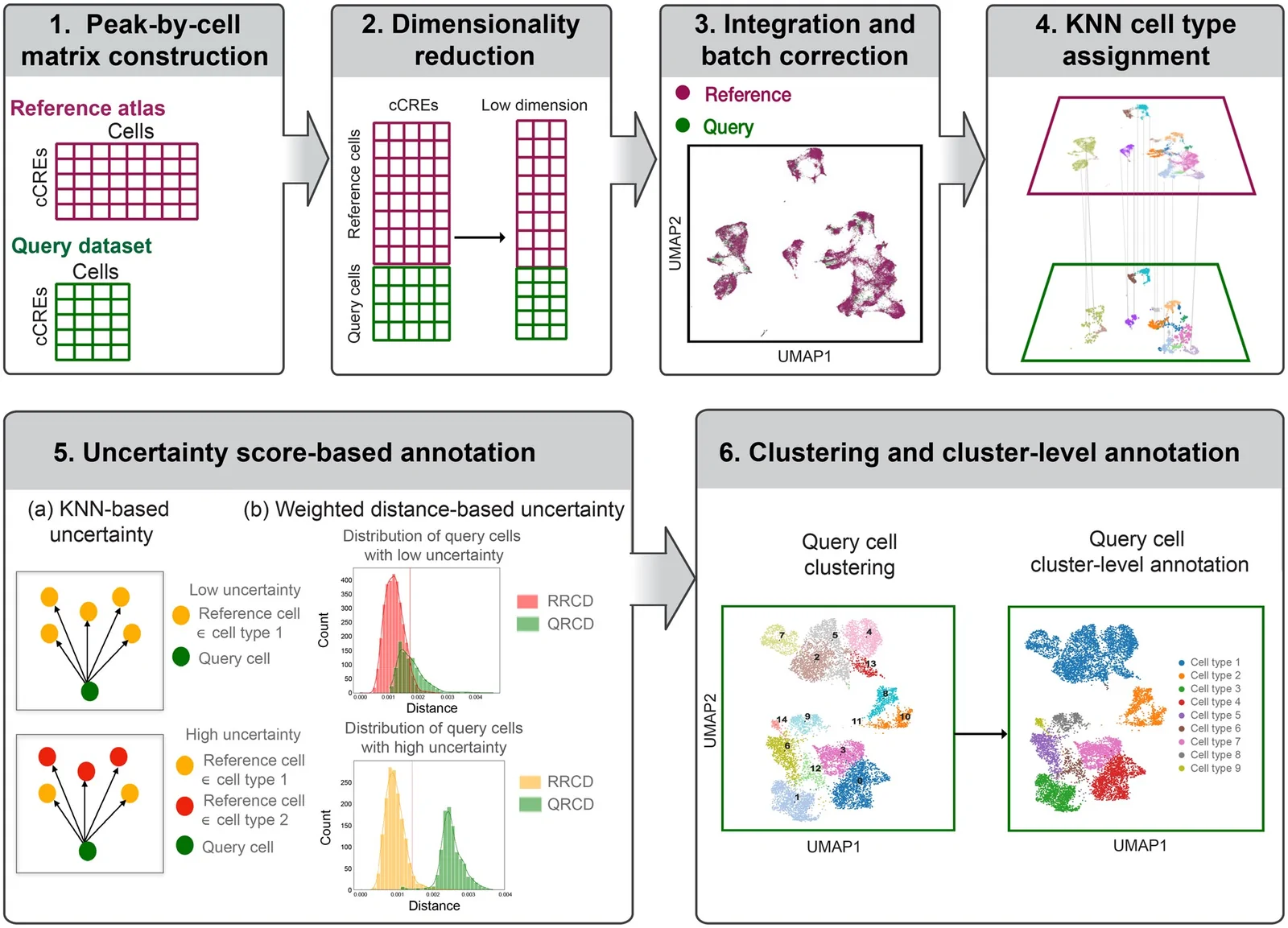
Schematic overview of scATAnno scATAnno consists of six steps. (1) Peak-by-cell matrix construction of reference atlas and query dataset. (2) Dimensionality reduction using spectral embedding. (3) Integration and batch correction. (4) KNN cell type assignment. The lower green plane shows query cells, each annotated using its closest neighbors in the reference cells in the upper purple plane. (5) Two uncertainty score measurements: KNN-based (a) and weighted distance-based (b). In the KNN-based uncertainty, a query cell is assigned a low uncertainty score if its K-nearest neighbors belong to the same reference cell type (top) or a high uncertainty score if its K-nearest neighbors belong to mixed reference cell types (bottom). In the weighted distance-based uncertainty, the distances of query cells to the centroid of each reference cell type (QRCD) are compared with the within-cell-type distribution of each reference cell type (RRCD). Query cells that fall within the 95th percentile of the within-cell-type distribution are assigned low uncertainty scores (top), and query cells that fall outside of the 95th percentile of the within-cell-type distribution are assigned high uncertainty scores (bottom). The red vertical lines represent the 95th percentile of RRCD distribution. (6) Clustering and annotation of query cells at the cell-cluster level. cCRE, candidate *cis*-regulatory element; KNN, k-nearest neighbors; QRCD, query-ref centroid distance; RRCD, ref-ref centroid distance; UMAP, Uniform Manifold Approximation and Projection.

scATAnno is a powerful automated tool for annotating cell types in scATAC-seq data. It takes fragment files of the query data and a reference atlas of choice as input, and predicts query cell types at both the individual cell and cell-cluster levels. As shown in **Figure 1**, scATAnno first constructs peak-by-cell matrices of the reference atlas and the query dataset. Reference atlases are generated from publicly available datasets, and the feature space is obtained either from the original publication [23] or through the MACS2 peak-calling algorithm [21,27]. If users want to build their own reference atlas data, it is easy to build customized reference atlases following the reference-building steps described in the Method section. Unlike scRNA-seq data, scATAC-seq data lack marker peaks, and thus the feature space varies between scATAC-seq datasets. To unify the feature space, scATAnno uses a set of peaks from the reference atlas and counts the reads from the query dataset that fall within these peaks, generating a peak-by-cell matrix for the query dataset. In step 2, matrices of reference and query cells are concatenated, followed by dimensionality reduction using spectral embedding. The workflow of scATAnno incorporates the SnapATAC2 tool to perform dimensionality reduction [29]. This converts the concatenated count matrix into a cell–cell similarity matrix and then performs eigenvector decomposition. SnapATAC2 [29] shows superior performance in dimensionality reduction, achieving shorter runtime, lower memory usage, and better clustering results than existing dimensionality reduction methods. We therefore chose this approach to reduce the dimensionality of the integrated reference and query data in our workflow [29,33]. In step 3, following the projection of reference and query cells into the same low-dimensional space, scATAnno optionally employs a batch effect removal method [30] to mitigate any potential batch effects, such as technical variations, between the reference and query data. In step 4, query cells are assigned cell labels using the KNN approach. Each query cell is annotated based on its k-nearest neighbors in the reference atlas. To measure the confidence of the KNN assignment, two distinct uncertainty score metrics are utilized in step 5. The first metric is the KNN-based uncertainty score, which has been described in previous publications [30,34]. This score is computed by considering the nearest neighbors of a query cell in the reference atlas. Query cells with nearest neighbors predominantly belonging to a single cell type are assigned low uncertainty scores, while those with neighbors consisting of a mixture of cell types receive higher uncertainty scores. While useful, the KNN-based uncertainty score has limitations and does not reflect the distance between a query cell and its assigned cell type cluster. This becomes a problem when query data contain an unknown cell population not represented in the reference, leading to inaccurate cell type assignment. To address this limitation, we introduced a weighted distance-based uncertainty score based on the intuition that query cells located far from the centroid of an assigned cell type represent an unknown population. The computation takes two sub-steps. First, we identified the centroids of reference cell types based on low-dimensional components, and hypothesized that low-dimensional components have varying discriminatory power for a specific cell type. Therefore, for each cell type, we assigned different weights to the components based on the closeness of reference cells to their respective cell type’s centroid along each dimension. Components with higher weights indicate greater importance in distinguishing a cell type. Second, a reference distance distribution is generated for each cell type by calculating the weighted distance between each reference cell and the centroid of its cell type. Subsequently, we computed the weighted distance of a query cell to the centroid of its assigned cell type and compared the query distance with the reference distance distribution. If the query-to-reference centroid distance is greater than the 95th percentile of the reference distance distribution, a query cell is assigned a high uncertainty score. The final uncertainty score for a query cell is determined by taking the maximum value between the KNN-based and weighted distance-based uncertainty scores. Query cells with high uncertainty scores are considered unknown cells. Finally, in step 6, query cells are clustered using the Leiden algorithm [28], and each cell cluster is annotated based on the most abundant cell type present in the cluster.

## Results

### scATAnno builds a reference atlas for healthy adult cells and achieves accurate cell type annotation

We first evaluated the performance of scATAnno by annotating cells within the reference dataset. We built a healthy adult reference atlas across 30 tissues from a publicly available dataset [23]. Specifically, we selected 100,158 deep-sequenced cells, and 890,130 adult-specific peaks provided by the original study (see Method). scATAnno built a ready-to-use reference atlas with 28 well-separated cell types (Figure S1A). To assess the accuracy of scATAnno in predicting cell types, we utilized cells generated from the original study but excluded from our reference building as query cells. scATAnno successfully integrated the query cells with the healthy adult reference atlas, as shown by UMAP (Figure S1B). We further visualized the predicted cell types of query cells along with their associated uncertainty scores (Figure S1C). Comparing the annotations from the original study (Figure S1D), scATAnno demonstrated accurate classification of most cells and displayed high uncertainty scores for individual cells situated in transition regions between cell clusters and peripheral regions of any cluster (Figure S1C). At the cluster-level annotation, scATAnno achieved an accuracy of 98.4% and an average F1 score of 98.2%. We also examined the impact of cell type similarities on performance by analyzing the accuracy and weighted F1 scores for each cell type. Notably, cell types that exhibited similarity to other cell types, such as ionic mesenchymal cells, displayed relatively lower accuracy and F1 scores (Figure S1E). Additionally, we compared the annotation performance using all peak regions *versus* promoter regions, and found that including all peak regions greatly improved the performance compared to using promoter regions alone. As shown in Figure S1F, we intersected all peak regions with TSS promoter regions extending to 1 kb, 2 kb, 5 kb, and 10 kb, and compared the annotation accuracy using extended promoter regions *versus* all regions. We observed that as the number of promoter regions increased, the accuracy also increased. Moreover, using all regions significantly enhanced the cell type annotation accuracy (98%) compared to the highest accuracy achieved using promoter regions extended to 10 kb (77%) (Figure S1F). Further breaking down the accuracy score of each cell type, we consistently found that using all regions achieved a higher accuracy score for each cell type than using promoter regions only (Figure S1G). Several cell types, such as ionic mesenchymal, keratinocyte, luteal, and perinucleolar compartment (PNC)-derived cells, consistently exhibited poor performance when using promoter regions only. This suggests that relying solely on promoter regions may fail to distinguish certain cell types.

Next, we investigated how accurately scATAnno detects an unknown cell population in query data not represented in the reference atlas. We explored the annotation in two scenarios: (1) ablating a cell type that is similar to other cell types, and (2) ablating a cell type distinct from other cell types (Figure S2A–C). We first ablated the vascular smooth muscle (VSM) cell type from the reference atlas to mimic the unknown cell type in the first scenario (Figure S2B). The original reference atlas (Figure S2A) demonstrates the close clustering of the VSM cell type with the mural cell type, indicating cell type similarity. Next, we showed step-by-step annotation results for query data visualized by UMAP (Figure S2D), highlighting the VSM cells in the query data within the dashed circle. We expected a high proportion of VSM cells in the query data to be unknown since this cell type was removed from the reference atlas. In the KNN assignment step, more than 80% of the VSM cells in the query data were assigned high uncertainty scores and successfully detected as unknown. This outcome could be attributed to the mixed KNN neighbors of these query cells. As most VSM query cells were detected by the KNN-based uncertainty score, the weighted distance-based uncertainty score did not significantly improve the results (Figure S2E). At the cluster level, all VSM cells in the query data were correctly annotated as unknown cells.

In the second scenario, we ablated the adrenal cortical cell type from the reference atlas (Figure S2C) and presented prediction results for the query data visualized by UMAP, highlighting the adrenal cortical cells in the query data in the dashed circle (Figure S2F). The adrenal cortical cell type is distinct from other cell types, as it is well-separated from any other cell type (Figure S2A). In the KNN assignment step, most adrenal cortical cells in the query data were incorrectly annotated as immune or mesenchymal cells with low uncertainty scores (Figure S2F). The misclassification is due to the fact that when the ablated cell type is distinct from other cell types, the KNN assignment erroneously assigns query cells to the next closest reference cluster while displaying low uncertainty scores. To overcome this limitation, we introduced the weighted distance-based uncertainty score. After applying this weighted distance-based uncertainty, we observed a significant increase in the proportion of expected unknown cells from 7.3% to 66.0%, and all adrenal cortical cells were correctly identified as unknown at the cluster level (Figure S2G). This demonstrates that the utilization of this more sophisticated weighted distance-based score results in a substantial improvement in the detection of unknown cell populations in the query data.

### scATAnno enables accurate classification of immune cell subtypes in PBMC Multiome data

We showcased the utility of scATAnno for integrating data from multiple datasets and cell type annotation by applying it to PBMC data. We built a large-scale PBMC reference atlas from publicly available data [21]. To construct this atlas, we downloaded raw FASTQ files to generate BAM files and fragment files across 15 healthy individuals and sampled cells. We then selected the immune cells (39,441 cells) and generated a reference peak set (344,492 peaks) by using MACS2 to call peaks (see Method). The UMAP plot displays a ready-to-use PBMC reference atlas encompassing 14 cell types (Figure S3A). To demonstrate the inter-dataset annotation performance of scATAnno, we downloaded PBMC 10k Multiome data from 10x Genomics as query cells. The advantage of using the Multiome data is the availability of both scATAC-seq and scRNA-seq profiles, which help validate the cell type annotation results using peak signals and gene expression patterns of canonical markers from the same cells. As shown in Figure S3B, scATAnno successfully integrated and removed the batch effects of the PBMC reference atlas and the query data.

To evaluate the performance of scATAnno, we compared its predicted cell types with those from Seurat (v3) [16], which first converts scATAC-seq data to gene activity scores and transfers labels from the scRNA-seq profile to scATAC-seq data. The Seurat (v3) annotation was directly obtained from the built-in data object stored in the Seurat function (see Method). Most cell types predicted by scATAnno were in agreement with those by Seurat (v3), except for the two B cell subtypes (Figure S3C–E). scATAnno annotates the lower B cell cluster as naive B and the upper cluster as memory B, while Seurat (v3) annotates the opposite. To validate the annotations produced by scATAnno, we leveraged the scRNA-seq profile and selected gene markers to distinguish the two B cell subtypes. Specifically, *CD27* and *TNFRSF13B* were selected as memory B cell markers, while *IGHM* and *TCL1A* were chosen as naive B cell markers [16,25]. As shown in Figure S3F, naive B cell markers (*IGHM* and *TCL1A*) exhibited increased expression levels in the cell cluster annotated as naive B, and memory B cell markers were upregulated in the cell cluster annotated as memory B, confirming the cell identities predicted by scATAnno. Lastly, scATAnno detected unknown cells not represented in the PBMC reference atlas. For example, a group of hematopoietic stem and progenitor (HSPC) cells annotated by Seurat (v3) was classified as unknown cells, due to the lack of this cell type in the PBMC reference atlas.

To further validate cell identities yielded by scATAnno, we examined the chromatin accessibility and gene expression of signature genes by plotting the peak signals using the scATAC-seq profile and gene expression distribution using the scRNA-seq profile. The coverage plot (Figure S3G) shows the normalized peak signal over a given genomic region ± 3 kb, while the ridge plot (Figure S3H) shows the distribution of normalized gene expression across cell groups. As shown in Figure S3G, the epigenetic features in the scATAC-seq profile displayed specific enrichment of peaks in corresponding cell types. For instance, T cell subtypes, including CD8^+^ T cells, CD4^+^ T cells, and regulatory T (Treg) cells, exhibited enrichment of peaks over the *CD3D* gene, which is a canonical marker of T cells. Specifically, CD8^+^ and CD4^+^ T cells showed enriched peak signals over the *CD8A* and *CD4* genomic regions, respectively. Tregs showed specific peak enrichment over the *FOXP3* locus, a gene marker for Treg cells. The gene expression patterns of signature genes in the scRNA-seq profile further confirmed the cell type annotation results (Figure S3H). For example, the *CD3D* gene, a marker for T cells, exhibited high expression levels in T cell subtypes. *CD8A* and *CD4* showed increased gene expression levels in the respective CD8^+^ and CD4^+^ T cells. *FOXP3* showed an increased expression level in the Treg population, although we observed more distinct gene activity in the scATAC-seq profile than in the scRNA-seq profile. Finally, *CD79A*, *GNLY*, *S100A9*, and *FLT3* showed strong peak signals and gene expression levels in B cells, natural killer (NK) cells, monocytes, and dendritic cells, respectively. The epigenetic features and gene expression patterns of signature genes support cell type annotation produced by scATAnno, demonstrating its robust capability in correctly identifying B cell subtypes misannotated by Seurat (v3).

### Benchmarking scATAnno against existing scATAC-seq cell typing methods

scATAnno possesses distinctive features for annotating scATAC-seq cell types compared to other existing methods, as outlined in **Table 2**. Only scATAnno, Cellcano [17], and Signac [17,26] are designed for scATAC-seq annotation, while Seurat, scJoint [35], and SingleR [9] are developed for scRNA-seq data. Notably, scATAnno is unique in its capability to harness enhancer signals, eliminating the need to translate peaks into proximal gene activity scores. For procedures that demand a gene activity score input, we adhered to each method’s guidelines to derive the gene activity score matrix. For tools intended for scRNA-seq data, the gene activity score was first procured from ArchR, followed by annotation based on a reference atlas and gene markers. Regarding uncertainty-aware mechanisms, scATAnno, Seurat, and Signac can yield uncertainty scores to predict unknown cells, while the other methods do not provide a means to quantify the reliability of their annotation results (Table 2). To benchmark scATAnno against Cellcano, scJoint, Signac, Seurat, and SingleR, we used two distinct datasets to evaluate performance. The first dataset was derived from a lymph node tumor of a patient diagnosed with B cell lymphoma, and the data can be downloaded from 10x Genomics. The second dataset featured PBMCs isolated through FACS (GSE123578).

**Table 2:** Comparison of features between scATAnno and other existing methods for annotating scATAC-seq cell types

Method	Version	Build for scATAC-seq annotation	Use distal enhancer signal	Do not need matched scRNA-seq	Provide uncertainty score	Predict unknown cells
scATAnno	1.0.0	√	√	√	√	√
Cellcano	1.0.2	√		√		
scJoint	1.0.0					
Seurat	4.3.0				√	√
Signac	1.9.0	√			√	√
SingleR	0.2.2					

*Note*: scATAC-seq, single-cell assay for transposase-accessible chromatin using sequencing; scRNA-seq, single-cell RNA sequencing.

**Figure 2:**
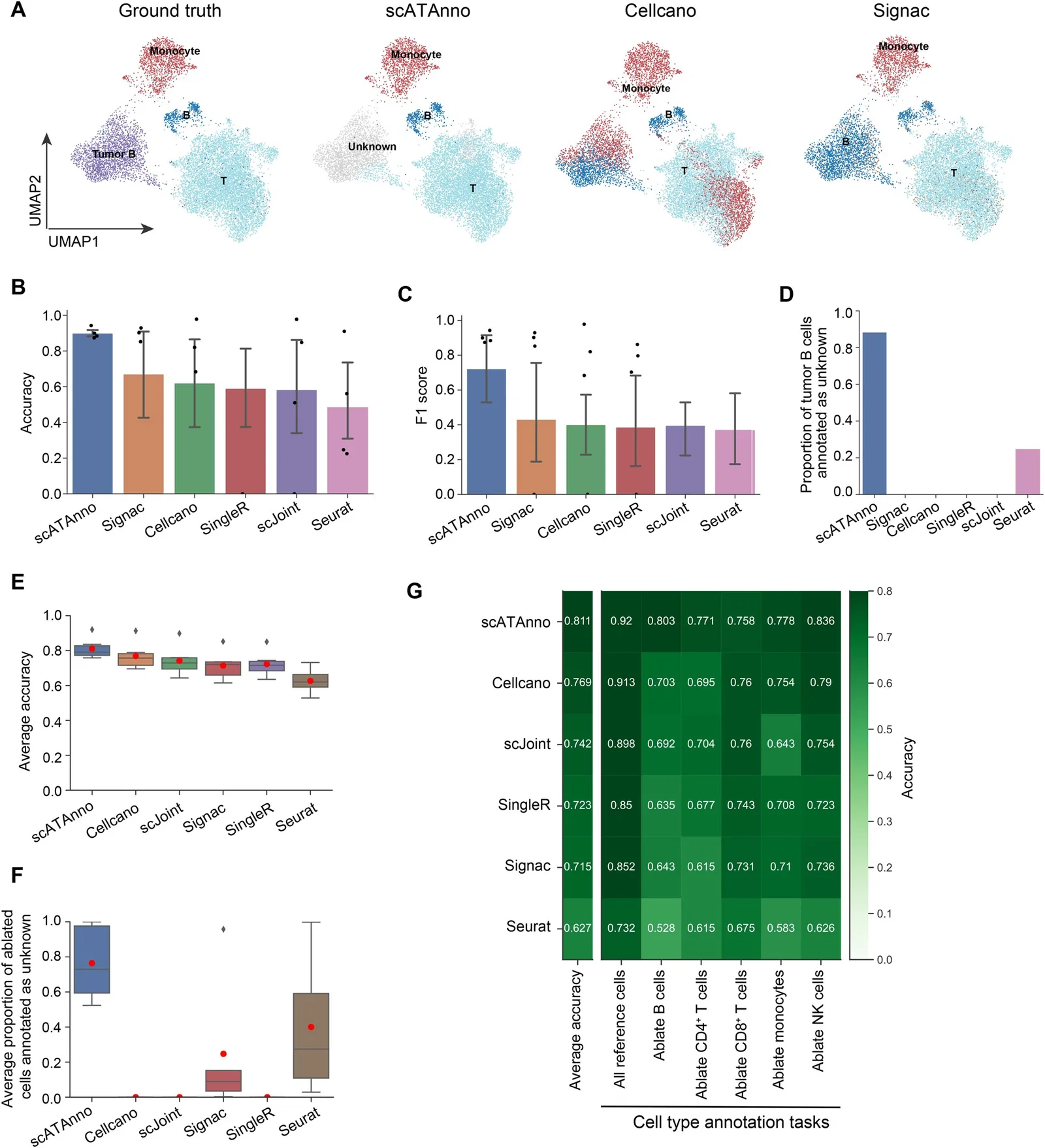
Benchmarking scATAnno against existing methods for cell type annotation **A**. UMAP representation of the ground truth cell labels of B cell lymphoma data, compared with cell type labels yielded by scATAnno, Cellcano, and Signac. **B**. Bar plot showing the accuracy scores of six methods (scATAnno, Cellcano, scJoint, Signac, Seurat, and SingleR) for B cell lymphoma annotation. **C**. Bar plot showing the F1 scores of six methods for B cell lymphoma annotation. **D**. Bar plot showing the proportion of tumor B cells correctly identified as unknown by each method. **E**. Bar plot showing the average accuracy scores of scATAnno, Cellcano, scJoint, Signac, Seurat, and SingleR for FACS-sorted PBMC data across six cell type annotation tasks (five ablation tasks and one task using all reference cells). **F**. Box plot showing the average proportion of ablated cells correctly identified as unknown in the PBMC data across five ablation tasks by each method. **G**. Heatmap showing the accuracy scores for six annotation tasks across methods. The five ablation scenarios involve removing one cell type at a time from the reference atlas: B cells, CD4^+^ T cells, CD8^+^ T cells, monocytes, and NK cells. The sixth task uses all reference cells. In (E and F), red dots indicate mean accuracy values and diamonds indicate outliers. FACS, fluorescence-activated cell sorting; PBMC, peripheral blood mononuclear cell; NK, natural killer.

We selected these datasets as they contained high-confidence cell type labels. We first benchmarked all methods on scATAC-seq data from a single-cell multi-omics dataset of a B cell lymphoma patient to investigate the performance of annotation in a more real-world disease context. This dataset has well-defined ground truth cell labels based on the matched scRNA-seq data from the same nuclei, and is composed of immune cells and tumor B cells. Previous research [36] revealed that tumor B cells from B cell lymphoma display thousands of differential chromatin accessibility peaks compared to normal B cells, highlighting substantial variations in chromatin architecture. Consequently, we regarded the true label of tumor B cells as unknown to distinguish them from normal B cells. We used the healthy PBMC reference atlas for all methods, with this B cell lymphoma dataset as a query. **Figure 2**A shows the UMAP plots of cells colored by cell types annotated by scATAnno, Cellcano, and Signac, respectively. Comparing annotations with ground truth labels, scATAnno successfully identified the majority of tumor B cells as unknown cells, whereas Cellcano annotated B cells as a mixed cluster of monocytes and B cells, and Signac failed to distinguish tumor B cells from normal B cells. Comparing accuracy scores across the six methods showed that scATAnno achieved the highest accuracy of 0.900, followed by Signac with an accuracy of 0.715 and Cellcano with an accuracy of 0.581 (Figure 2B). scATAnno significantly outperformed the other methods in this case study (Figure 2C). In this regard, unlike other conventional methods that classified B cell lymphoma cells as B cells, scATAnno correctly identified the lymphoma cells as a distinct cell population, which is consistent with the scRNA-seq analysis (Figure 2D).

Our second benchmark used the FACS-isolated PBMC data as query data since this dataset has very high-quality cell types. We downloaded the raw fragment files, used Signac to filter out low-quality cells, and obtained 15,643 immune cells composed of CD8^+^ T cells, CD4^+^ T cells, B cells, monocytes, and NK cells. We applied all annotation methods to this dataset and compared them with the ground truth labels determined by cell sorting. We created six annotation tasks by using all reference cells or systematically removing each cell type (CD8^+^ T cells, CD4^+^ T cells, B cells, monocytes, and NK cells) from the PBMC reference atlas to test whether ablations of cell types in the reference atlas would affect the performance of annotation. Compared to the average accuracy score across methods, scATAnno achieved the best average accuracy score of 0.811; Cellcano was the second best with an average accuracy of 0.769; and scJoint was the third with an average accuracy of 0.742 (Figure 2E–G). scATAnno significantly outperformed the other methods in the ablation study, and it was able to identify the unknown cell population ablated from the reference atlas (Figure 2F). To evaluate computational efficiency, we benchmarked the performance of scATAnno on PBMC scATAC-seq datasets ranging from 1000 to 110,000 cells. As shown in Figure S4A and B, scATAnno exhibited shorter running times and a linear increase in computation time as the number of annotated cells increased. However, due to its peak-based approach, scATAnno’s memory usage was comparable to that of other peak-based methods, such as Signac, but higher than that of gene activity score-based methods. Furthermore, we tested scATAnno’s performance on query datasets with 5, 10, and 50 rare B (CD19^+^) cells. The annotation results, shown in Figure S4C, demonstrated its effectiveness in accurately identifying rare cell populations. We conclude that scATAnno performs best in terms of accuracy and running times, and is the most robust method for cell type annotation in PBMC-sorted data.

### scATAnno enables multi-layer cell type annotation of tumor samples

Cancer samples typically consist of tumor cells and immune cells. To identify tumor cells and immune cells in the tumor microenvironment (TME), we next showed the utility of combinations of reference atlases including both healthy adult cell and cancer cell atlases to enable multi-layer cell type annotation in the following two steps.

#### Identifying cancer cell clusters and normal cells in the TME

**Figure 3:**
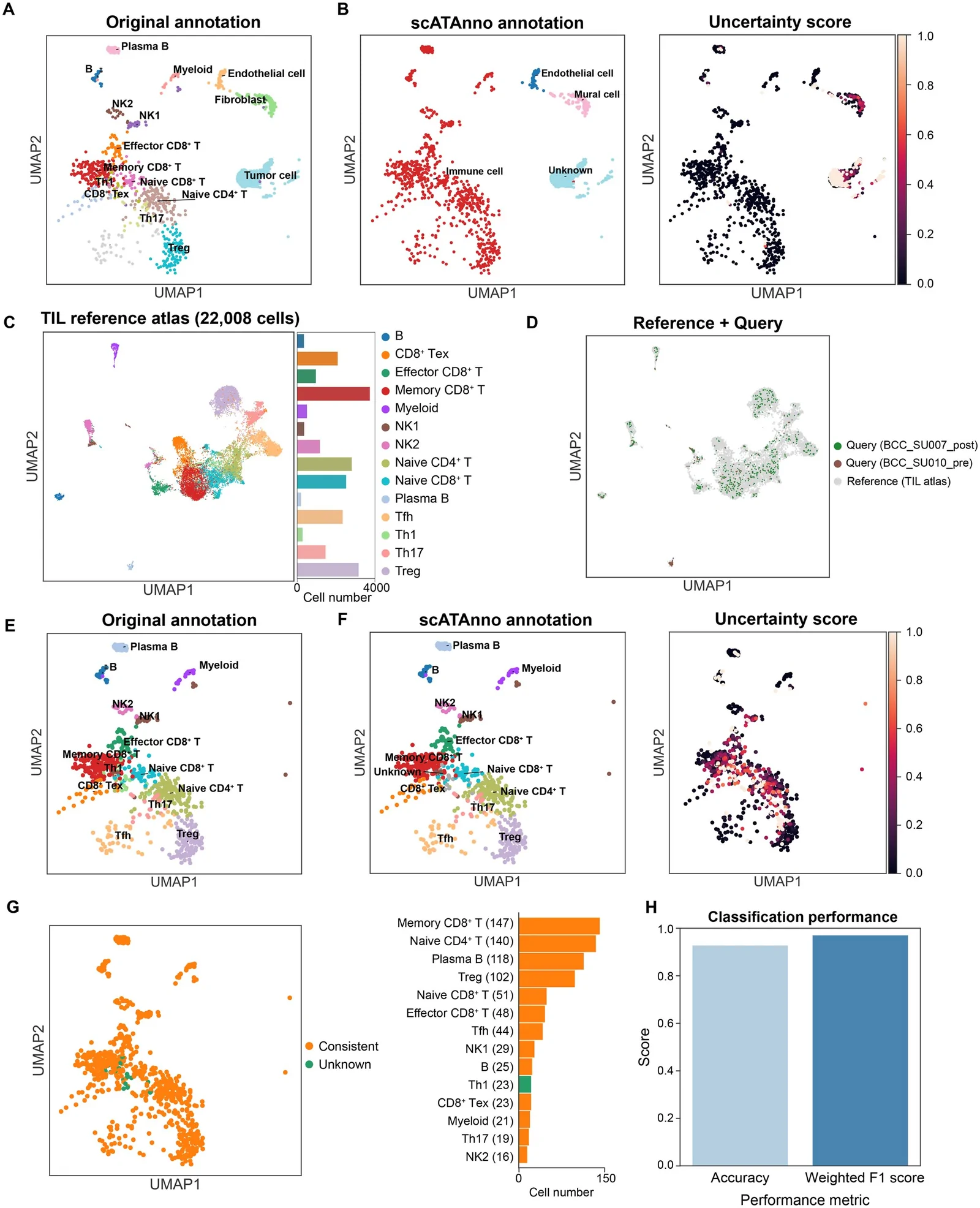
BCC case study **A**. UMAP representation of query cells colored by cell types from the original publication (Satpathy et al. [21]). **B**. UMAP representation of query cells colored by cell types predicted by scATAnno (left) and the distribution of uncertainty scores (right). **C**. Construction of the BCC TIL reference atlas. **D**. Integration of immune cells from two holdout BCC samples with the TIL reference atlas. **E**. UMAP representation of immune cells from the query data colored by cell types from the original publication (Satpathy et al. [21]). **F**. UMAP representation of immune cells colored by cell types predicted by scATAnno (left) and the distribution of uncertainty scores (right). **G**. Comparison of cell type annotations between scATAnno and the original study, shown by a UMAP plot (left) and a stacked bar plot of cell counts (right). The stacked bar plot displays the number of consistently annotated or unknown cells per cell type. **H**. Classification accuracy and weighted F1 score. Th1, T helper 1; Th17, T helper 17; Tex, exhausted T; Tfh, T follicular helper; Treg, regulatory T; BCC, basal cell carcinoma; TIL, tumor-infiltrating lymphocyte.

In the first step, we used scATAnno to distinguish tumor cells from immune cells in cancer samples using a healthy adult reference atlas. We selected two BCC samples held out from the BCC TIL reference building as query data and mapped them to the healthy adult reference atlas. The BCC samples were pre- or post-treated by immunotherapy and comprised a diverse ecosystem of cell types in the TME. To balance the treatment conditions, we selected one pre-treatment and one post-treatment sample as the query. The query data contained tumor cells that were not present in the healthy adult reference atlas; therefore, intuitively, the tumor cells should display high uncertainty scores and be identified as unknown. As shown in Figure S5A–C, query cells were successfully integrated with the reference atlas. By scrutinizing the predicted cell types and uncertainty score distribution on the UMAP space of query cells (**Figure 3**A and B), we observed that tumor cells showed high uncertainty scores and were accurately annotated as unknown at the cluster level. Furthermore, scATAnno accurately annotated endothelial and immune cells with low uncertainty scores (Figure 3B). Fibroblasts, on the other hand, were identified as mural cells, also known as pericytes [23,37], which could be a result of the strong connection between pericytes and fibroblasts in the TME [37,38]. The chromatin accessibility profile confirmed the enrichment of peak signals over *PDGFRB*, an important marker of pericytes [39], in the mural group (Figure S5D). scATAnno can leverage the healthy adult reference atlas to annotate cell types in tumor samples and accurately identify normal cells and immune cells while annotating tumor cells as unknown.

#### Annotating TIL subtypes in the TME

In the second step, to better characterize the subtypes of TILs, we extracted the immune cells detected above using a healthy adult reference atlas and applied a TIL reference atlas from BCC to produce a more fine-grained annotation of immune cells. To build the BCC TIL reference, we obtained fragment files of 17 BCC samples from raw FASTQ files [21] and generated a reference peak set (344,492 peaks) using MACS2 peaks (see Method). The immune cells from the two BCC query samples were well integrated with the TIL reference data (Figure 3C and D), and most of them were accurately annotated by scATAnno (Figure 3E–H). By comparing the original annotation (Figure 3E) with the scATAnno annotation (Figure 3F), only a small group of T helper 1 (Th1) cells was annotated as unknown and exhibited a high-level uncertainty score. These Th1 cells were located in the transition region in the UMAP representation, which could lead to high uncertainty scores (Figure 3F, right). The remaining cells showed annotation results consistent with the original annotation. Overall, scATAnno achieved an accuracy of 92.9% and a weighted F1 score of 97.1%. To further confirm the TIL subtypes yielded by scATAnno, we inspected the chromatin accessibility of signature genes. Each cell type exhibited enhanced levels of accessibility over the signature genes (Figure S5D). For example, the enrichment of peak signals at the *PDCD1* locus, which is a T cell exhaustion signature, confirmed the identity of the CD8^+^ exhausted T cell (Tex) group. The unique accessibility in the *FOXP3* locus validated the Treg cell identity. Taken together, these findings demonstrate that scATAnno provides robust multi-layer annotation for tumor samples. It first identifies the coarse level of cell type annotation using the healthy adult reference and detects the immune cells in the TME, and then classifies a broad spectrum of TIL subtypes of immune cells.

Overall, scATAnno not only accurately classifies cell types, but also robustly enables cross-condition cell type annotation. We believe that scATAnno can be useful for annotating scATAC-seq data under various conditions.

### scATAnno leverages a TIL atlas of BCC to accurately predict TIL cells in TNBC

**Figure 4:**
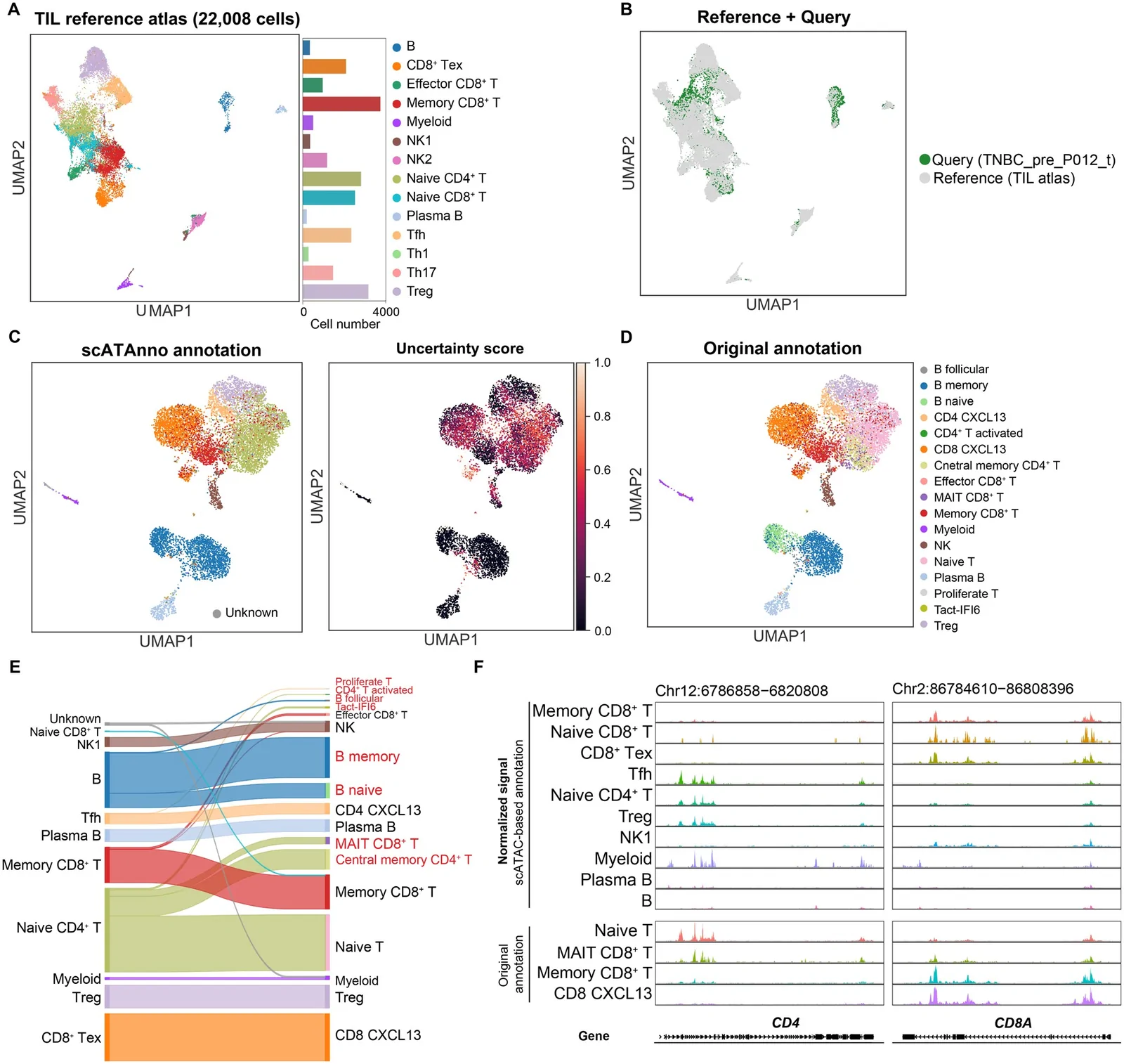
TNBC case study **A**. Construction of the BCC TIL reference atlas. **B**. Integration of TNBC query data with the TIL reference atlas. **C**. UMAP representation of query cells colored by cell types predicted by scATAnno (left) and the distribution of uncertainty scores (right). **D**. UMAP representation of query cells colored by cell types from the original publication (Zhang et al. [22]). **E**. Sankey plot mapping cell types annotated by scATAnno (left) to cell types annotated in the original study (right). Cell types with different names or those not represented in the TIL reference atlas are highlighted in red. **F**. Coverage plots of chromatin accessibility over the *CD4* and *CD8A* regions across cell types predicted by scATAnno (top) and across the four subtypes selected from the original study (bottom). TNBC, triple-negative breast cancer.

We demonstrated that scATAnno can be applied to more complex systems, such as biopsy samples from cancer patients, and capture cellular heterogeneity of tumor-infiltrating immune cells in the TME. We applied the TIL reference atlas from BCC samples to annotate TIL cells in a TNBC cohort [22]. To test scATAnno, we used a TNBC sample comprising 9935 cells as query data. As shown in **Figure 4**A and B, scATAnno built a TIL reference atlas encompassing 14 cell types in the UMAP space and successfully integrated the TNBC query data with the TIL reference atlas. To evaluate the performance of scATAnno, we compared the predicted query cell types (Figure 4C) with those in the original study [22], which annotated scATAC-seq cells by transferring the labels from the matching scRNA-seq profile (Figure 4D). The Sankey plot in Figure 4E compares the labels yielded by scATAnno (left) to those obtained from the original study (right). The cell types predicted by the original study but not represented in the BCC TIL reference atlas are highlighted in red. Most cell types annotated by scATAnno were in line with those in the original study. For example, B cells predicted by scATAnno were annotated as B memory, B naive, and B follicular cells in the original study. CD8^+^ Tex is a Tex group annotated as CD8 CXCL13 in the original study, which confirms the exhaustion features in this cell group [22]. T follicular helper (Tfh) is originally annotated as CD4 CXCL13, which has Tfh features according to the original study [22]. The cell type annotation result can be further validated with enhanced accessibility of signature genes (Figure S6).

We next investigated the inconsistent annotations and validated our results by examining the accessibility near genomic regions of signature genes across cell types. A group of naive CD4^+^ T cells was annotated as a mixture of naive T, MAIT CD8^+^ T, and central memory CD4^+^ T cells in the original publication [22]. Central memory CD4^+^ T cells were not represented in the BCC TIL reference atlas; hence, this cell type could not be predicted by scATAnno. To investigate the cell identity of originally annotated MAIT CD8^+^ T cells, we examined the peak enrichment of MAIT CD8^+^ T cells near the *CD4* and *CD8A* loci, and found that this group of cells exhibited stronger enrichment of peaks over the *CD4* locus than over the *CD8A* locus. Moreover, peak signals over the *CD4* and *CD8A* loci across four cell types yielded by the original study revealed a resemblance between MAIT CD8^+^ T and naive CD4^+^ T cells, rather than the other CD8^+^ T cell groups (Figure 4F). Therefore, we conclude that cells labeled as MAIT CD8^+^ T cells are more likely CD4^+^ T cells, as inferred by scATAnno, rather than CD8^+^ T cells. Collectively, these observations demonstrate that scATAnno is a powerful tool where one can use a TIL atlas from one cancer type to identify TILs from a different cancer type, which greatly facilitates the use of scATAC-seq data to study TIL subtypes under complex disease conditions.

## Discussion

scATAnno is a cell type annotation tool specifically designed for single-cell chromatin accessibility data. It directly annotates new scATAC-seq data, eliminating the need for matching scRNA-seq profiling. Unlike many existing methods, scATAnno does not rely solely on promoters and nearby regions to convert peaks into gene activity scores. Instead, it incorporates a significant number of enhancers, regardless of distance from promoter and gene body regions, providing a more comprehensive understanding of the regulatory landscape and significantly improving the characterization of cell type-specific accessibility patterns.

scATAnno enables the generation of scATAC-seq-based reference atlases from publicly available datasets and efficiently annotates cell types of newly generated query data using fragment files. Importantly, scATAnno incorporates KNN-based and weighted distance-based uncertainty scores to assess the confidence of cell type assignments and detect unknown cell populations in the query data. The novel quantification of the weighted distance uncertainty score greatly improves the accuracy of identifying unknown cells in the query data. Furthermore, scATAnno supports both single-reference atlas annotation for less complex query samples, such as PBMCs, and multi-atlas annotation for samples with greater complexity.

scATAnno exhibits a strong capability of cell type annotation at the scATAC-seq level. In the PBMC case study, scATAnno builds a PBMC reference and improves annotation performance by correcting cells mislabeled by Seurat (v3). In the TNBC study, scATAnno successfully identifies TIL subtypes in TNBC using a BCC TIL reference. In the BCC study, scATAnno develops two-round annotation tasks and shows robust cross-condition annotation using a healthy adult reference to annotate cells within the TME. Furthermore, the scATAnno workflow can be extended to any data, allowing for reference building and query annotation beyond the presented cases.

There are limitations of scATAnno that require further development. Currently, it supports scATAC-seq data mapped to the human reference genome. Future improvements aim to support different reference genome versions, such as mouse genomes. Additionally, the scope of cell types in the current reference atlases may be incomplete, resulting in the absence of some cell types using scATAnno. Moreover, while scATAnno captures the heterogeneity of most TIL subtypes in the TNBC data using a BCC TIL atlas, it has not been tested for annotating scATAC-seq data from other cancer types. To address these limitations, we seek to incorporate more diverse atlases in the future. The rapid expansion of scATAC-seq data from multiple species and diverse systems will fill gaps in our reference sets and improve the capabilities of scATAnno, ultimately enhancing our understanding of chromatin configurations underlying cell states in complex biological systems.

## Code availability

scATAnno code is available at GitHub (https://github.com/Yijia-Jiang/scATAnno-main). The code has also been submitted to BioCode at the National Genomics Data Center (NGDC), China National Center for Bioinformation (CNCB) (BioCode: BT007771), which is publicly accessible at https://ngdc.cncb.ac.cn/biocode/tool/7771. The ReadTheDocs tutorial for scATAnno, along with the processed reference atlases and query data for download, is available at https://scatanno-main.readthedocs.io.

## CRediT author statement

**Yijia Jiang:** Conceptualization, Data curation, Methodology, Software, Visualization, Writing – original draft. **Zhirui Hu:** Methodology, Writing – original draft. **Feng Lu:** Methodology, Software, Visualization, Validation. **Allen W. Lynch:** Software. **Junchen Jiang:** Formal analysis, Visualization. **Alexander Zhu:** Formal analysis, Visualization. **Ziqi Zeng:** Formal analysis, Validation. **Yi Zhang:** Writing – original draft. **Gongwei Wu:** Writing – original draft. **Yingtian Xie:** Resources. **Rong Li:** Resources. **Ningxuan Zhou:** Resources. **Cliff A. Meyer:** Resources. **Paloma Cejas:** Resources. **Myles Brown:** Project administration, Supervision. **Henry W. Long:** Project administration, Supervision, Writing – review & editing. **Xintao Qiu:** Conceptualization, Methodology, Software, Visualization, Project administration, Supervision, Writing – review & editing. All authors have read and approved the final manuscript.

## Competing interests

Myles Brown is a consultant to and serves on the Scientific Advisory Board (SAB) of Novartis, from which he also receives sponsored research support. Myles Brown serves on the SAB of Kronos Bio and has equity in the company. Myles Brown serves on the SAB of GV20 Therapeutics and has equity in the company. Myles Brown serves on the SAB of FibroGen. Henry W. Long receives research funding from Novartis. All the other authors have declared no competing interests.

## Supplementary Material

qzaf108_Supplementary_Data

## ORCID

0000-0002-0239-6999 (Yijia Jiang)

0000-0001-6352-4854 (Zhirui Hu)

0009-0002-4460-4503 (Feng Lu)

0000-0003-0796-3232 (Allen W. Lynch)

0009-0002-7259-2896 (Junchen Jiang)

0009-0003-1042-7710 (Alexander Zhu)

0009-0004-1774-6148 (Ziqi Zeng)

0000-0002-7453-6188 (Yi Zhang)

0000-0002-2509-2381 (Gongwei Wu)

0000-0003-0206-457X (Yingtian Xie)

0009-0000-1046-9580 (Rong Li)

0000-0002-7623-2276 (Ningxuan Zhou)

0000-0002-8321-2839 (Cliff A. Meyer)

0000-0002-2090-7748 (Paloma Cejas)

0000-0002-8213-1658 (Myles Brown)

0000-0001-6849-6629 (Henry W. Long)

0000-0002-8560-7017 (Xintao Qiu)
