# Supplementary material for: scATAnno: Automated Cell Type Annotation for Single-cell ATAC-seq Data
Source: Genomics Proteomics Bioinformatics. 2025 Nov 24;24(2):qzaf108. doi: 10.1093/gpbjnl/qzaf108 (PMC13568823; doi:10.1093/gpbjnl/qzaf108)
Supplement: Supplementary file 1 [file qzaf108_supplementary_data.zip › supplementary material captions.docx]

**Figure S1 Cell type annotation of holdout healthy adult cells**

**A.** Construction of healthy adult reference atlas. **B.** Integration of holdout healthy adult cells and the reference atlas. **C.** UMAP representation of query cells annotated by original publication (Sapathy *et al*.). **D.** UMAP representation of query cells colored by cell types and uncertainty score distribution produced by scATAnno. **E.** Computation of F1 score (left) and accuracy (right) of each cell type. **F.** Bar plot of average accuracy scores of using promoter regions (extending 1 kb, 2 kb, 5 kb, 10 kb) and all peak regions. Overall accuracy scores are 3.37%, 34.2%, 58.3%, 77.2%, and 98.2%, respectively. **G.** Box plot of accuracy scores breaking down by each cell type using promoter regions (extending 1 kb, 2 kb, 5 kb, 10 kb) and all peak regions. Each color dot shows the accuracy score of a cell type shown in the legend.

**Figure S2 Simulation of annotating holdout healthy adult cells using ablated reference atlas**

**A.** Healthy adult reference atlas using all reference cells highlighting vascular smooth cells and adrenal cortical cells. **B.** Construction of a new healthy adult reference atlas ablated vascular smooth cells from the original atlas. **C.** Construction of a new healthy adult reference atlas ablated adrenal cortical cell type. **D.** Query cells on UMAP representation, highlighting vascular smooth cells in a dashed circle. The arrow points to the three-step annotation results for the vascular smooth cells: KNN assignment, weighted distance-based assignment, and cluster-level assignment. **E.** Proportion of correctly identifying vascular smooth as unknown cells in each assignment step. Proportion is computed as the number of unknown cells divided by the total number of vascular smooth cells in the query data. **F.** Query cells on UMAP representation, highlighting adrenal cortical cells in a dashed circle and three-step annotation results. **G.** Proportion of correctly identifying adrenal cortical as unknown cells in each assignment step, following the same computation as (E).

**Figure S3 PBMC case study**

**A.** Constructed PBMC reference atlas. **B.** Integration of PBMC query data and the reference atlas. **C.** UMAP representation of query cells colored by cell types predicted from scATAnno (left) and distribution of uncertainty score (right). **D.** UMAP representation of query cells colored by cell types predicted from Seurat annotation. **E.** Comparisons of cell type annotation from scATAnno and Seurat shown by UMAP (left), and by stacked bar plot of predicted cell types (right). The stacked bar plot displays the counts of consistently annotated cells, inconsistently annotated cells, and unknown cells for each cell type. **F.** Gene expression levels of signature markers for B cell subtypes using scRNA-profile. **G.** Coverage plots of chromatin accessibility over signature genes across predicted cell types: *CD3D* for T cells, *CD8A* for CD8 T, *CD4* for CD4 T, *FOXP3* for Treg, *CD79A* for B cells, *GNLY* for NK, *S100A9* for monocytes, and *FLT3* for cDC and pDC. **H.** Ridge plots of the gene expression levels of signature genes

**Figure S4**  **Benchmark**

**A.** Running time requirement, benchmark against other methods for 1 k to 110 k PBMC scATAC-seq cells annotation. **B.** Memory requirements, benchmark against other methods for 1 k to 110 k PBMC scATAC-seq cells annotation. **C.** Annotation results of 5, 10, and 50 query B (CD19) cells using scATAnno.

**Figure S5**  **Coverage plots of BCC TIL study**

**A.** Construction of Healthy Adult reference atlas. **B.** Integration of two holdout BCC samples and healthy reference atlas. **C.** Uncertainty score of query cells visualized in the integrated UMAP. **D.** Coverage plots of BCC case study. Each plot shows the chromatin accessibility of a signature gene across cell types. The cell types are obtained from the combination of two-round annotations. Chromatin accessibility of the *CD3D* locus is enriched for T cells, and *CD8A* and *CD4* loci are enriched for CD8 T and CD4 cells respectively. Chromatin accessibility of *PDCD1* enriched for CD8 TEx and Tfh, and chromatin accessibility of *FOXP3* enriched for Treg. Chromatin accessibility of *GNLY* enriched for NK, *S100A9* enriched for myeloid, *PECAM1* enriched for endothelial cells. Chromatin accessibility of *CD79A* is enriched for B cells, and *CD27* is enriched for plasma B cells. Chromatin accessibility of *PDGFRB* is enriched for mural cells.

**Figure S6 Coverage plots of TNBC case study**

Each plot shows the chromatin accessibility of a signature gene across predicted cell types. Chromatin accessibility of *CD3D* locus is enriched for T cells, chromatin accessibility of *FOXP3* enriched for Treg, chromatin accessibility of *PDCD1* enriched for CD8 TEx and Tfh, chromatin accessibility of *GNLY* enriched for NK, chromatin accessibility of *CCR7* enriched for naive T cells, chromatin accessibility *CXCR5* enriched for Tfh, chromatin accessibility of *S100A9* enriched for myeloid, chromatin accessibility of *CD79A* enriched for B cells and *CD27* enriched for plasma B cells.
