# Supplementary material for: scATAnno: Automated Cell Type Annotation for Single-cell ATAC-seq Data
Source: Genomics Proteomics Bioinformatics. 2025 Nov 24;24(2):qzaf108. doi: 10.1093/gpbjnl/qzaf108 (PMC13568823; doi:10.1093/gpbjnl/qzaf108)
Supplement: Supplementary file 1 [file qzaf108_supplementary_data.zip › Figure S1.pdf]

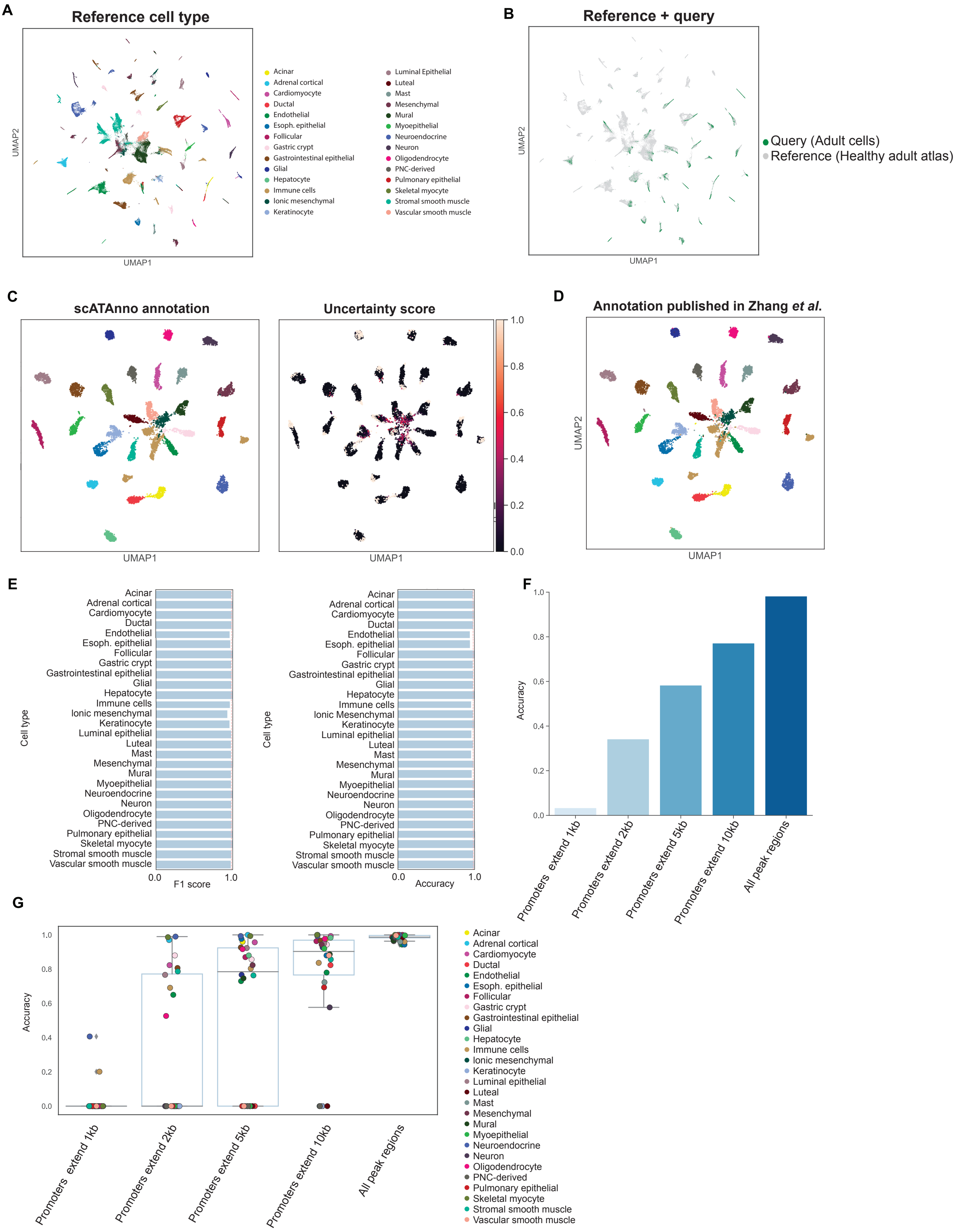
A
Reference cell type
UMAP2
UMAP1
Acinar
Adrenal cortical
Cardiomyocyte
Ductal
Endothelial
Esoph. epithelial
Follicular
Gastric crypt
Gastrointestinal epithelial
Glial
Hepatocyte
Immune cells
Ionic mesenchymal
Keratinocyte
Luminal Epithelial
Luteal
Mast
Mesenchymal
Mural
Myoepithelial
Neuroendocrine
Neuron
Oligodendrocyte
PNC-derived
Pulmonary epithelial
Skeletal myocyte
Stromal smooth muscle
Vascular smooth muscle
B
Reference + query
UMAP2
UMAP1
Query (Adult cells)
Reference (Healthy adult atlas)
C
scATAnno annotation
UMAP1
Uncertainty score
UMAP1
1.0
0.8
0.6
0.4
0.2
0.0
D
Annotation published in Zhang et al.
UMAP2
UMAP1
E
Cell type
F1 score
Accuracy
0.0
1.0
Ionic Mesenchymal
Luminal epithelial
F
Accuracy
0.0
0.2
0.4
0.6
0.8
1.0
Promoters extend 1kb
Promoters extend 2kb
Promoters extend 5kb
Promoters extend 10kb
All peak regions
G
Accuracy
0.0
0.2
0.4
0.6
0.8
1.0
Promoters extend 1kb
Promoters extend 2kb
Promoters extend 5kb
Promoters extend 10kb
All peak regions
