# Supplementary material for: scATAnno: Automated Cell Type Annotation for Single-cell ATAC-seq Data
Source: Genomics Proteomics Bioinformatics. 2025 Nov 24;24(2):qzaf108. doi: 10.1093/gpbjnl/qzaf108 (PMC13568823; doi:10.1093/gpbjnl/qzaf108)
Supplement: Supplementary file 1 [file qzaf108_supplementary_data.zip › Figure S2.pdf]

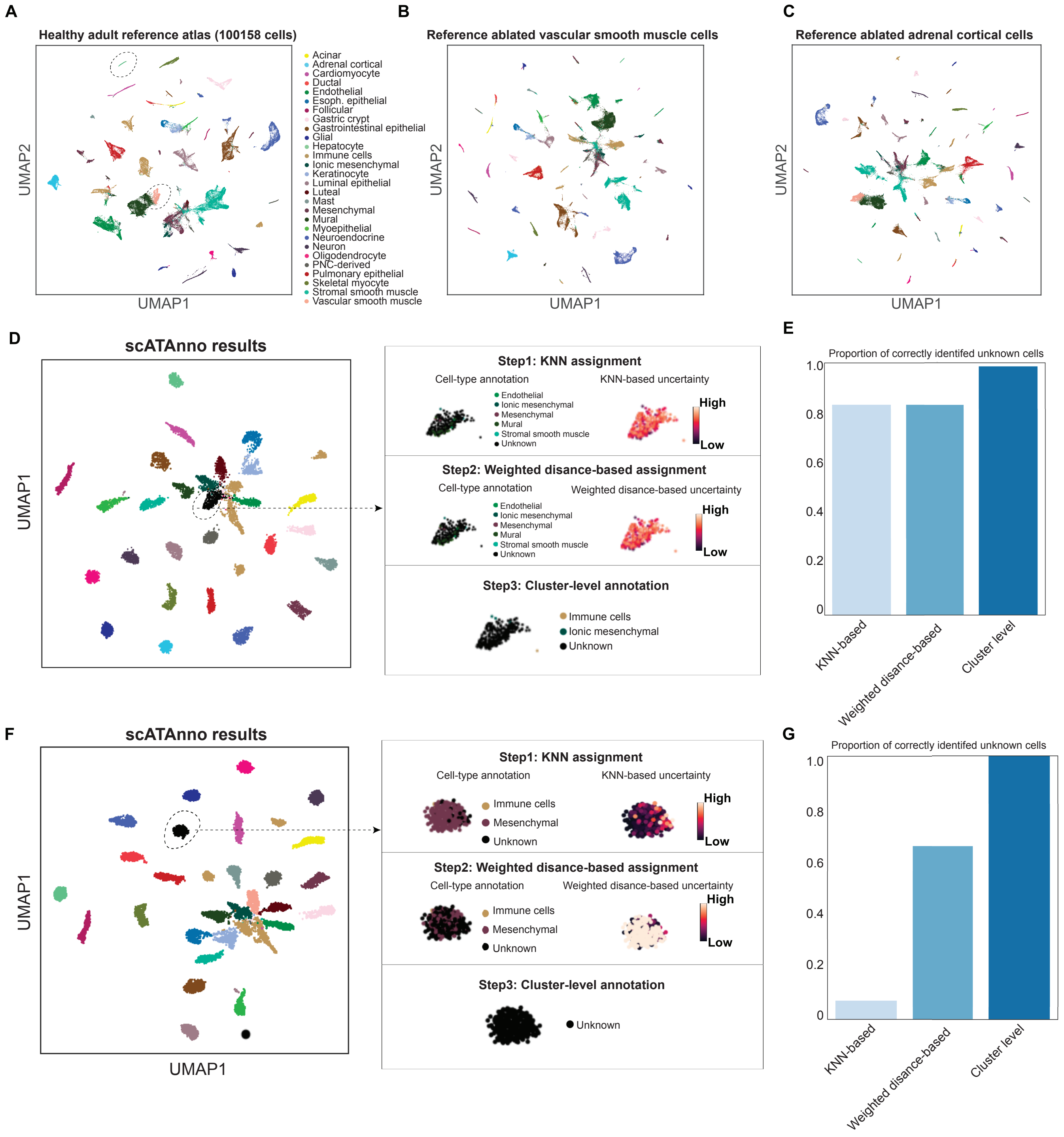

A
Healthy adult reference atlas (100158 cells)
UMAP2
UMAP1
Acinar
Adrenal cortical
Cardiomyocyte
Ductal
Endothelial
Esoph. epithelial
Follicular
Gastric crypt
Gastrointestinal epithelial
Glial
Hepatocyte
Immune cells
Ionic mesenchymal
Keratinocyte
Luminal epithelial
Luteal
Mast
Mesenchymal
Mural
Myoepithelial
Neuroendocrine
Neuron
Oligodendrocyte
PNC-derived
Pulmonary epithelial
Skeletal myocyte
Stromal smooth muscle
Vascular smooth muscle
B
Reference ablated vascular smooth muscle cells
UMAP2
UMAP1
C
Reference ablated adrenal cortical cells
UMAP2
UMAP1
D
scATAnno results
UMAP1
Step1: KNN assignment
Cell-type annotation
KNN-based uncertainty
Endothelial
Ionic mesenchymal
Mesenchymal
Mural
Stromal smooth muscle
Unknown
High
Low
Step2: Weighted disance-based assignment
Cell-type annotation
Weighted disance-based uncertainty
Endothelial
Ionic mesenchymal
Mesenchymal
Mural
Stromal smooth muscle
Unknown
High
Low
Step3: Cluster-level annotation
Immune cells
Ionic mesenchymal
Unknown
E
Proportion of correctly identifed unknown cells
1.0
0.8
0.6
0.4
0.2
0
KNN-based
Weighted disance-based
Cluster level
F
scATAnno results
UMAP1
UMAP1
Step1: KNN assignment
Cell-type annotation
KNN-based uncertainty
Immune cells
Mesenchymal
Unknown
High
Low
Step2: Weighted disance-based assignment
Cell-type annotation
Weighted disance-based uncertainty
Immune cells
Mesenchymal
Unknown
High
Low
Step3: Cluster-level annotation
Unknown
G
Proportion of correctly identifed unknown cells
1.0
0.8
0.6
0.4
0.2
0
KNN-based
Weighted disance-based
Cluster level
