# Supplementary material for: scATAnno: Automated Cell Type Annotation for Single-cell ATAC-seq Data
Source: Genomics Proteomics Bioinformatics. 2025 Nov 24;24(2):qzaf108. doi: 10.1093/gpbjnl/qzaf108 (PMC13568823; doi:10.1093/gpbjnl/qzaf108)
Supplement: Supplementary file 1 [file qzaf108_supplementary_data.zip › Figure S3.pdf]

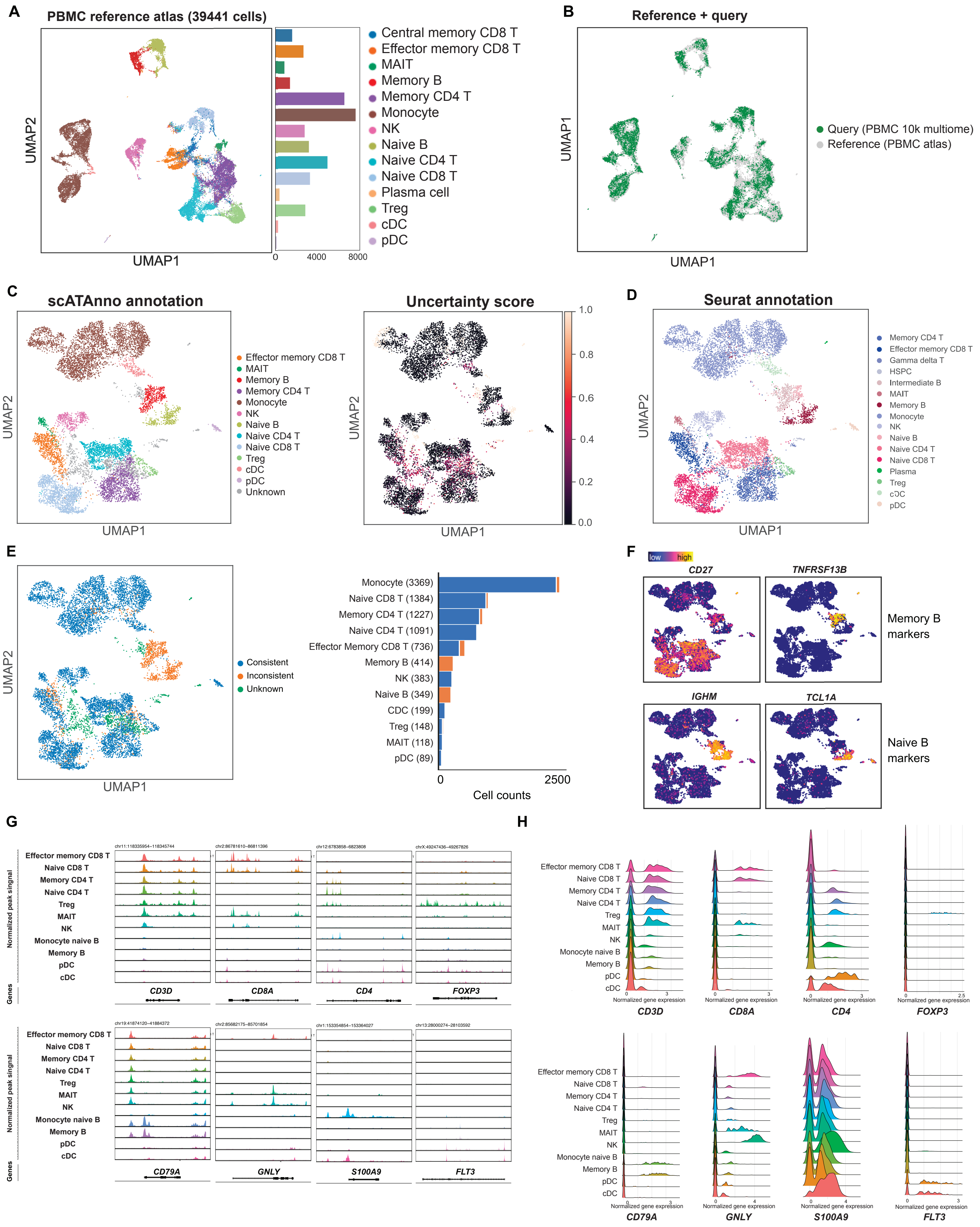

A
PBMC reference atlas (39441 cells)
UMAP2
UMAP1
0
4000
8000
Central memory CD8 T
Effector memory CD8 T
MAIT
Memory B
Memory CD4 T
Monocyte
NK
Naive B
Naive CD4 T
Naive CD8 T
Plasma cell
Treg
cDC
pDC
B
Reference + query
UMAP1
UMAP1
Query (PBMC 10k multiome)
Reference (PBMC atlas)
C
scATAnno annotation
UMAP2
UMAP1
Effector memory CD8 T
MAIT
Memory B
Memory CD4 T
Monocyte
NK
Naive B
Naive CD4 T
Naive CD8 T
Treg
cDC
pDC
Unknown
Uncertainty score
UMAP2
UMAP1
1.0
0.8
0.6
0.4
0.2
0.0
D
Seurat annotation
UMAP2
UMAP1
Memory CD4 T
Effector memory CD8 T
Gamma delta T
HSPC
Intermediate B
MAIT
Memory B
Monocyte
NK
Naive B
Naive CD4 T
Naive CD8 T
Plasma
Treg
cDC
pDC
E
UMAP2
UMAP1
Consistent
Inconsistent
Unknown
Monocyte (3369)
Naive CD8 T (1384)
Memory CD4 T (1227)
Naive CD4 T (1091)
Effector Memory CD8 T (736)
Memory B (414)
NK (383)
Naive B (349)
CDC (199)
Treg (148)
MAIT (118)
pDC (89)
0
2500
Cell counts
F
low
high
CD27
TNFRSF13B
Memory B markers
IGHM
TCL1A
Naive B markers
G
Normalized peak singnal
Genes
Effector memory CD8 T
Naive CD8 T
Memory CD4 T
Naive CD4 T
Treg
MAIT
NK
Monocyte naive B
Memory B
pDC
cDC
CD3D
CD8A
CD4
FOXP3
CD79A
GNLY
S100A9
FLT3
H
Effector memory CD8 T
Naive CD8 T
Memory CD4 T
Naive CD4 T
Treg
MAIT
NK
Monocyte naive B
Memory B
pDC
cDC
Normalized gene expression
CD3D
CD8A
CD4
FOXP3
CD79A
GNLY
S100A9
FLT3
