# Supplementary material for: scATAnno: Automated Cell Type Annotation for Single-cell ATAC-seq Data
Source: Genomics Proteomics Bioinformatics. 2025 Nov 24;24(2):qzaf108. doi: 10.1093/gpbjnl/qzaf108 (PMC13568823; doi:10.1093/gpbjnl/qzaf108)
Supplement: Supplementary file 1 [file qzaf108_supplementary_data.zip › Figure S4.pdf]

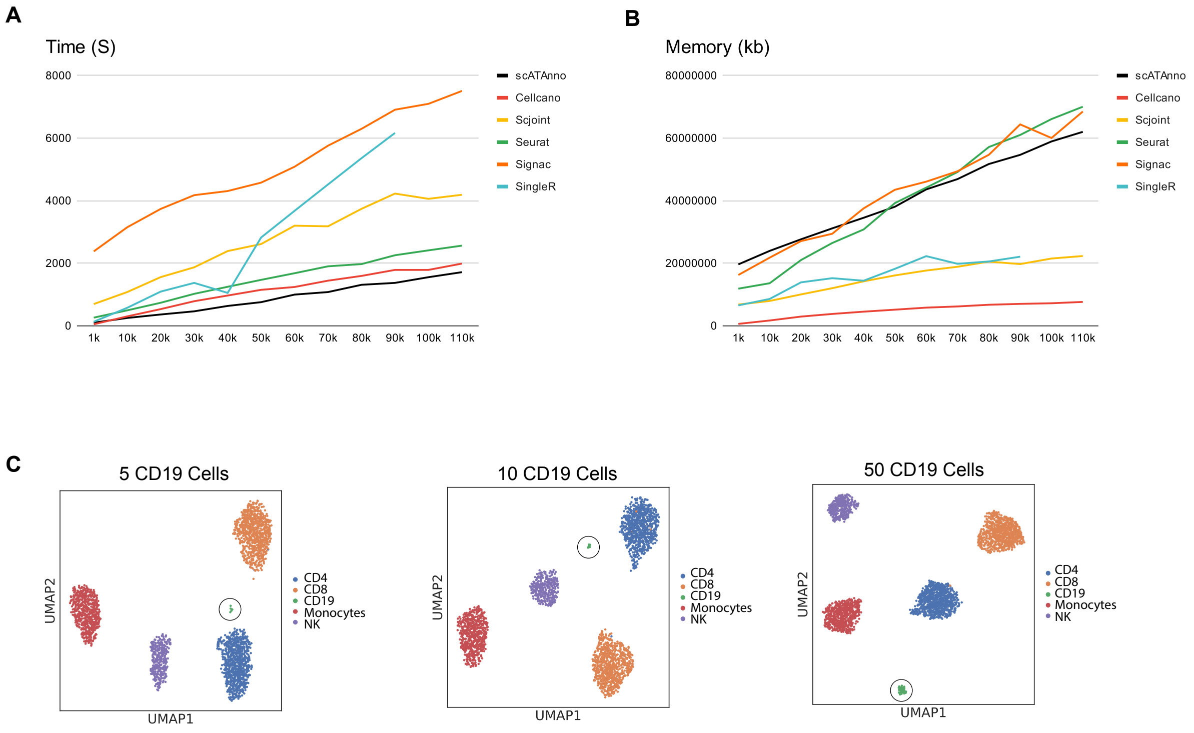

A
Time (S)
8000
6000
4000
2000
0
1k
10k
20k
30k
40k
50k
60k
70k
80k
90k
100k
110k
scATAnno
Cellcano
Scjoint
Seurat
Signac
SingleR
B
Memory (kb)
80000000
60000000
40000000
20000000
0
1k
10k
20k
30k
40k
50k
60k
70k
80k
90k
100k
110k
scATAnno
Cellcano
Scjoint
Seurat
Signac
SingleR
C
5 CD19 Cells
UMAP2
UMAP1
CD4
CD8
CD19
Monocytes
NK
10 CD19 Cells
UMAP2
UMAP1
CD4
CD8
CD19
Monocytes
NK
50 CD19 Cells
UMAP2
UMAP1
CD4
CD8
CD19
Monocytes
NK
