# Supplementary material for: scATAnno: Automated Cell Type Annotation for Single-cell ATAC-seq Data
Source: Genomics Proteomics Bioinformatics. 2025 Nov 24;24(2):qzaf108. doi: 10.1093/gpbjnl/qzaf108 (PMC13568823; doi:10.1093/gpbjnl/qzaf108)
Supplement: Supplementary file 1 [file qzaf108_supplementary_data.zip › Figure S5.pdf]

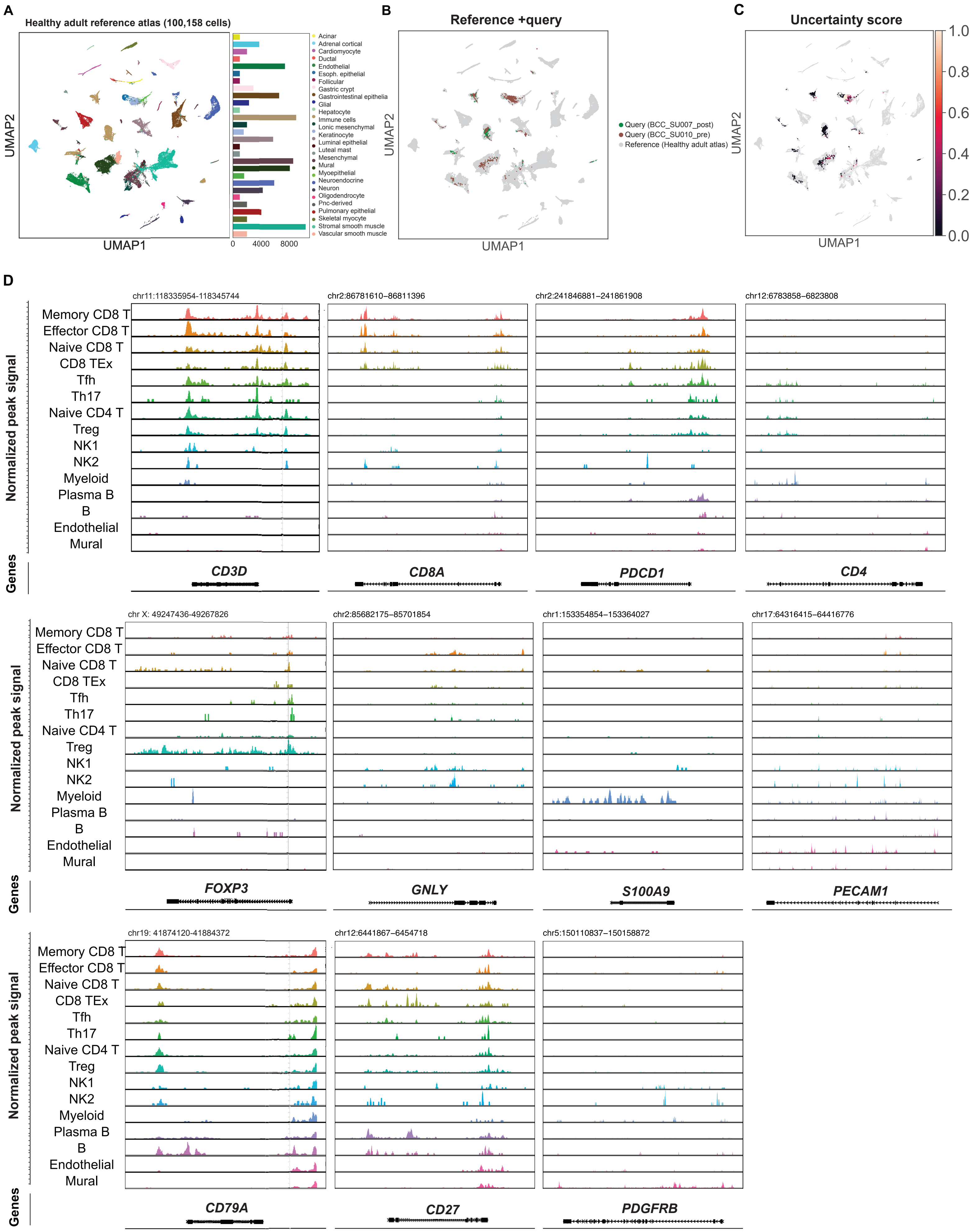
A
Healthy adult reference atlas (100,158 cells)
UMAP2
UMAP1
0
4000
8000
Acinar
Adrenal cortical
Cardiomyocyte
Ductal
Endothelial
Esoph. epithelial
Follicular
Gastric crypt
Gastrointestinal epithelia
Glial
Hepatocyte
Immune cells
Lonic mesenchymal
Keratinocyte
Luminal epithelial
Luteal mast
Mesenchymal
Mural
Myoepithelial
Neuroendocrine
Neuron
Oligodendrocyte
Pnc-derived
Pulmonary epithelial
Skeletal myocyte
Stromal smooth muscle
Vascular smooth muscle
B
Reference +query
UMAP2
UMAP1
Query (BCC_SU007_post)
Query (BCC_SU010_pre)
Reference (Healthy adult atlas)
C
Uncertainty score
UMAP2
UMAP1
1.0
0.8
0.6
0.4
0.2
0.0
D
Normalized peak signal
Memory CD8 T
Effector CD8 T
Naive CD8 T
CD8 TEx
Tfh
Th17
Naive CD4 T
Treg
NK1
NK2
Myeloid
Plasma B
B
Endothelial
Mural
Genes
chr11:118335954-118345744
chr2:86781610–86811396
chr2:241846881–241861908
chr12:6783858–6823808
CD3D
CD8A
PDCD1
CD4
chr X: 49247436-49267826
chr2:85682175–85701854
chr1:153354854–153364027
chr17:64316415–64416776
FOXP3
GNLY
S100A9
PECAM1
chr19: 41874120-41884372
chr12:6441867–6454718
chr5:150110837–150158872
CD79A
CD27
PDGFRB
