# Supplementary material for: scATAnno: Automated Cell Type Annotation for Single-cell ATAC-seq Data
Source: Genomics Proteomics Bioinformatics. 2025 Nov 24;24(2):qzaf108. doi: 10.1093/gpbjnl/qzaf108 (PMC13568823; doi:10.1093/gpbjnl/qzaf108)
Supplement: Supplementary file 1 [file qzaf108_supplementary_data.zip › Figure S6.pdf]

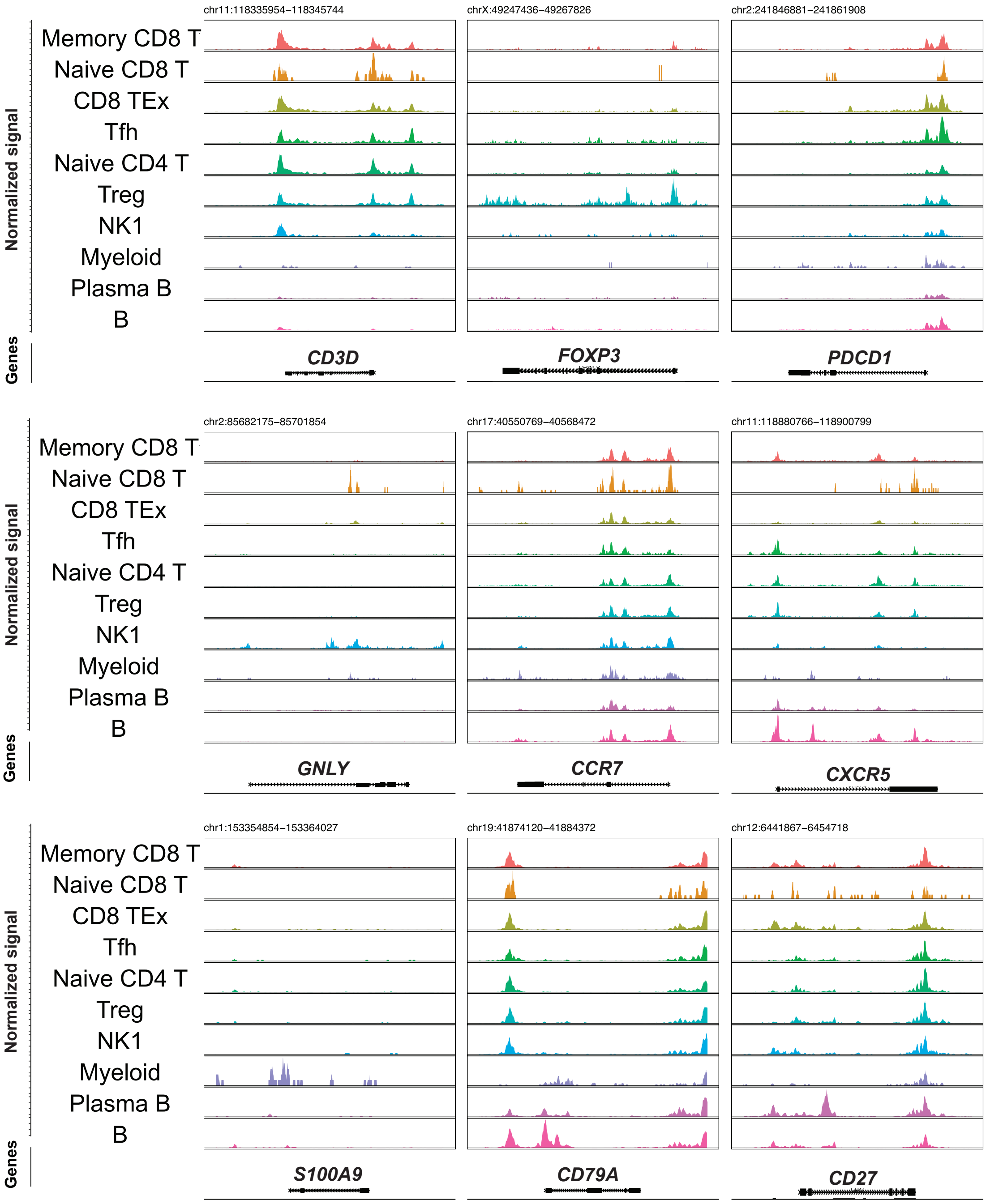
chr11:118335954–118345744
chrX:49247436–49267826
chr2:241846881–241861908
Memory CD8 T
Naive CD8 T
CD8 TEx
Tfh
Naive CD4 T
Treg
NK1
Myeloid
Plasma B
B
Normalized signal
Genes
CD3D
FOXP3
PDCD1
chr2:85682175–85701854
chr17:40550769–40568472
chr11:118880766–118900799
GNLY
CCR7
CXCR5
chr1:153354854–153364027
chr19:41874120–41884372
chr12:6441867–6454718
S100A9
CD79A
CD27
